# The Plasmidomic Landscape of Clinical Methicillin-Resistant *Staphylococcus aureus* Isolates from Malaysia

**DOI:** 10.3390/antibiotics12040733

**Published:** 2023-04-09

**Authors:** Esra’a I. Al-Trad, Ching Hoong Chew, Ainal Mardziah Che Hamzah, Zarizal Suhaili, Nor Iza A. Rahman, Salwani Ismail, Suat Moi Puah, Kek Heng Chua, Stephen M. Kwong, Chew Chieng Yeo

**Affiliations:** 1 Centre for Research in Infectious Diseases and Biotechnology (CeRIDB), Faculty of Medicine, Universiti Sultan Zainal Abidin, Kuala Terengganu 20400, Malaysia; 2 Faculty of Health Sciences, Universiti Sultan Zainal Abidin, Kuala Nerus 21300, Malaysia; 3 Faculty of Bioresources and Food Industry, Universiti Sultan Zainal Abidin, Besut 22200, Malaysia; 4 Department of Biomedical Science, Faculty of Medicine, Universiti Malaya, Kuala Lumpur 50603, Malaysia; 5 Infectious Diseases & Microbiology, School of Medicine, Western Sydney University, Campbelltown 2560, Australia

**Keywords:** multidrug resistant MRSA, plasmids, plasmid mobilization, replicon, resistance, whole genome sequencing

## Abstract

Methicillin-resistant *Staphylococcus aureus* (MRSA) is a priority nosocomial pathogen with plasmids playing a crucial role in its genetic adaptability, particularly in the acquisition and spread of antimicrobial resistance. In this study, the genome sequences of 79 MSRA clinical isolates from Terengganu, Malaysia, (obtained between 2016 and 2020) along with an additional 15 Malaysian MRSA genomes from GenBank were analyzed for their plasmid content. The majority (90%, 85/94) of the Malaysian MRSA isolates harbored 1–4 plasmids each. In total, 189 plasmid sequences were identified ranging in size from 2.3 kb to ca. 58 kb, spanning all seven distinctive plasmid replication initiator (replicase) types. Resistance genes (either to antimicrobials, heavy metals, and/or biocides) were found in 74% (140/189) of these plasmids. Small plasmids (<5 kb) were predominant (63.5%, 120/189) with a RepL replicase plasmid harboring the *ermC* gene that confers resistance to macrolides, lincosamides, and streptogramin B (MLS_B_) identified in 63 MRSA isolates. A low carriage of conjugative plasmids was observed (*n* = 2), but the majority (64.5%, 122/189) of the non-conjugative plasmids have mobilizable potential. The results obtained enabled us to gain a rare view of the plasmidomic landscape of Malaysian MRSA isolates and reinforces their importance in the evolution of this pathogen.

## 1. Introduction

*Staphylococcus aureus* is a Gram-positive bacterium that normally resides as a commensal in the anterior nares and on the skin and mucous membranes of healthy individuals. However, *S. aureus* can cause a wide spectrum of infections that can potentially be lethal in immunocompromised patients, children, and the elderly [1,2,3]. *S. aureus* clinical isolates showed increasing trends of resistance to several antimicrobial agents, heavy metals, and disinfectants due mainly to the horizontal acquisition of mobile elements such as plasmids, transposons and the staphylococcal chromosomal cassette (SCC) element, or by mutations in chromosomal genes [2,4]. The prominent resistant strain of *S. aureus* is methicillin-resistant *S. aureus* (MRSA), which exhibits resistance against most β-lactam antibiotics and is often multidrug-resistant (MDR) (i.e., displays resistance to three or more classes of antimicrobials) [3]. MRSA has been listed as one of the priority pathogens in urgent need of new antimicrobials by the World Health Organization (WHO) in 2017 [5]. Asia has one of the highest prevalences of MRSA [6,7,8], with MDR MRSA being endemic in most Asian hospitals [9,10]. However, the prevalence varies significantly between countries and has altered markedly over time [11,12]. In Malaysia, the MRSA prevalence rates in public hospitals were estimated at 14.9% in 2020 but dropped to 7% in 2021 [13]. Unfortunately, data on the molecular genotyping of MRSA isolates from Malaysia is scarce, with very few published papers on their clonal types and diversity. Recent reviews of the literature and data from Malaysian MRSA isolates appear to indicate the predominance of strains of clonal complex (CC), i.e., CC8, CC1, and CC22 lineages [14,15]. 

Plasmids replicate independently of the chromosome and in some instances can be integrated into the chromosome [16,17]. The carriage of plasmids has been used in tracking the dissemination of antibiotic resistance genes and in the epidemiological surveillance of bacterial disease outbreaks [18,19,20]. Like most plasmids, staphylococcal plasmids exhibit diversity in their components that initiate replication as well as in their transmission mechanisms (conjugation or mobilization) [21]. Plasmids also carry variable genes that are important for bacterial adaptability and survival under some conditions (such as exposure to antibiotics or heavy metals) [22]. Previous analyses of *S. aureus* plasmids indicate that the plasmid size is strongly correlated with the mode of replication used [23]. The rolling circle replication mechanism is used by plasmids that are smaller than 10 kb, whereas those larger than 14.5 kb normally utilize the theta-mode of replication. Plasmids between 10 and 14.5 kb can utilize either of these replication mechanisms [23]. Conjugative and mobilizable plasmids play an important role in horizontal gene transfer, providing surrounding bacteria with access to a reservoir of shared genes, and thus facilitating the transmission and evolution of a multidrug resistance [24,25]. Interestingly, some staphylococcal plasmids that were previously thought to be non-mobilizable were found to be mobilized through the carriage of pWBG749-like *oriT* mimic sequences. Plasmids harboring these *oriT* mimics can be mobilized in *trans* by pWBG749-family plasmids that encode the appropriate relaxasome accessory factor, SmpO [26,27]. Sequences that resemble the pSK41 *oriT* sequences have also been found in numerous staphylococcal plasmids [28,29], inferring that the relaxase in *trans* mechanism for conjugative mobilization is widespread in staphylococcal plasmids. 

Despite the wide availability of the genome sequences of *S. aureus* in public databases [30,31], there have been few published reports of the genome sequences of MRSA isolates from Malaysia as well as entries in the databases [14,15]. No detailed and comprehensive molecular characterizations of plasmids from Malaysian MRSA isolates have been reported. This study thus aims to fill in this essential gap in our knowledge by identifying and characterizing plasmids from the whole genome sequences of 79 MRSA clinical isolates that were obtained from a tertiary hospital in the state of Terengganu, Malaysia, from 2016 to 2020. The genome sequences of 15 other Malaysian clinical MRSA isolates (2009–2016) that were available in the databases were also included in this analysis. Together, this has given us an important first view of the plasmidomic landscape of MRSA in Malaysia. This paper focuses on the plasmids of these Malaysian isolates and in-depth analyses of their genome sequences; characteristics and evolutionary relationships will be the subject of a future manuscript.

## 2. Results and Discussion

### 2.1. Detection and Distribution of Plasmids in the Malaysian MRSA Isolates

All the 79 isolates from HSNZ were resistant to cefoxitin and were validated to be MRSA by positive PCR-amplification of the *nucA* and *mecA* genes as detailed in the Materials and Methods (Section 3.2) prior to whole genome sequencing.

**Figure 1:**
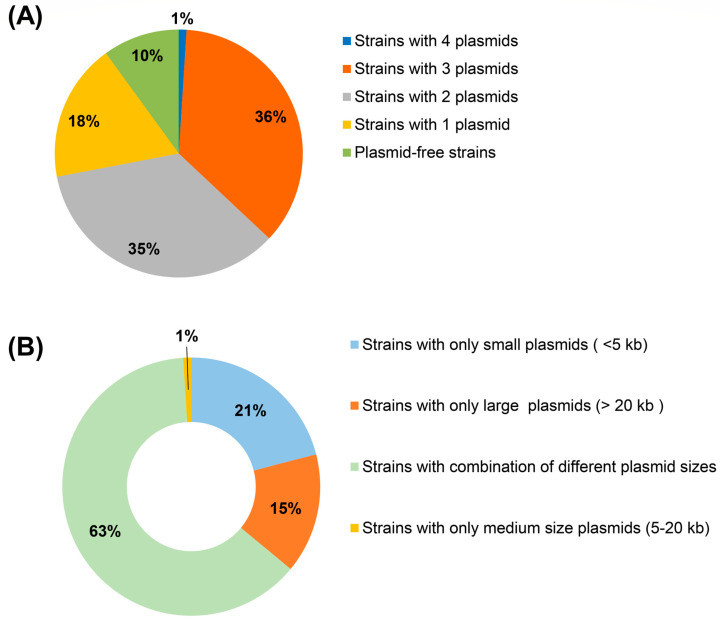
Distribution of plasmids in the Malaysian MRSA isolates. (**A**) The number of plasmids detected in each sequenced MRSA isolate. (**B**) The distribution of plasmids in each sequenced MRSA isolate based on the plasmid size.

Analysis of the WGS data for the 94 Malaysian MRSA isolates, i.e., 79 from HSNZ that were sequenced in this study and 15 that were obtained from the database (refer to Supplementary Table S1 for accession numbers and their background details), revealed that 90% (85/94) of the isolates carried plasmids while the remaining 10% (9/94) appeared to be plasmid-free isolates (Figure 1A). Three of these plasmid-free isolates were sequenced in the present study while the remaining six isolates were obtained from the GenBank database. The high prevalence of isolates harboring plasmids was in agreement with an earlier study where 89% of MRSA isolates (*n* = 73) from the Czech Republic were found to carry plasmids [32] and other studies which also report a similarly high prevalence of plasmids (in the range of 70–85%) in *S. aureus* [33,34]. The number of plasmids present in this study varied from one to four plasmids per isolate (Figure 1), on which 18% (17/94) of the isolates harbored one plasmid, while the existence of multiple plasmids within the same isolate was detected in 72% (68/94) of the isolates and were distributed as follows: 36% (34/94) of the isolates contained three plasmids, 35% (33/94) contained two plasmids, and 1% (1/94) likely harbored four plasmids (Figure 1A). Co-carriage of several plasmids in a single isolate is in agreement with previous studies [35,36], where up to eight plasmids in a single *S. aureus* isolate have been reported [37,38]. The high prevalence of plasmids may provide a selective advantage for MRSA isolates despite the metabolic burden that their carriage may impose. This is because plasmids facilitate the transmission of genetic information, and thus play a central role in the adaptation and survival of *Staphylococcus* species in challenging environments [39,40,41]. 

A total of 173 plasmids were identified from the whole genome sequence data of 76 plasmid-carrying isolates from HSNZ that were sequenced in the present study. Another 16 plasmids were detected from the genome sequence of nine other Malaysian MRSA isolates from GenBank (Supplementary Table S1). With that said, the total number of plasmids identified from the sequenced Malaysian MRSA isolates was 189, and the size ranged from 2.3 kb to 58 kb. This plasmid size range fit with previous results where staphylococcal plasmids were estimated to vary in size from 1 to >60 kb [38,42], and plasmids larger than 60 kb were uncommon [38]. In an earlier study using plasmid DNA extraction followed by restriction enzyme digestion, a total of 24 different plasmid profiles were determined from 73 MRSA isolates with sizes that ranged from 1.3 to 55 kb [32].

In terms of size, about 21% (18/85) of plasmid-carrying isolates harbored only small-sized plasmids, which were less than 5 kb, while 15% (13/85) only carried large plasmids greater than 20 kb, with the largest being ca. 58.4 kb (found in SauR23). One MRSA isolate, SauR262, carried a medium-sized plasmid of around 15 kb (1%, or 1/85) and the remaining isolates (63%, or 53/85) contained plasmid combinations of different size ranges (Figure 1B).

### 2.2. Distribution of Plasmid Replicon Types

Plasmids from Gram-positive bacteria (including staphylococci) can be classified into different groups based on the replication initiation (replicase) proteins that they possess [43,44], and this scheme was incorporated into the PlasmidFinder database [45,46]. However, the identification of additional replicon types makes for discordant classification of some staphylococcal plasmids [23], and we believe that primary classification should be based on the conserved domains of the replicases [42]. Among the identified plasmid sequences in this study, 72% (137/189) of them were single-replicon, whereas 28% (52/189) plasmids appeared to have two or more complete *rep* genes belonging to different families. Such multi-replicon staphylococcal plasmids had been previously documented [47,48], and they potentially have an increased host range, making them efficient vectors for the transmission of antibiotic resistance and virulence genes between distantly related strains or even genera [48,49,50,51]. Nevertheless, some of the replicases of these multi-replicon plasmids were truncated and likely non-functional, possibly being remnants of past co-integration events with smaller plasmids.

**Figure 2:**
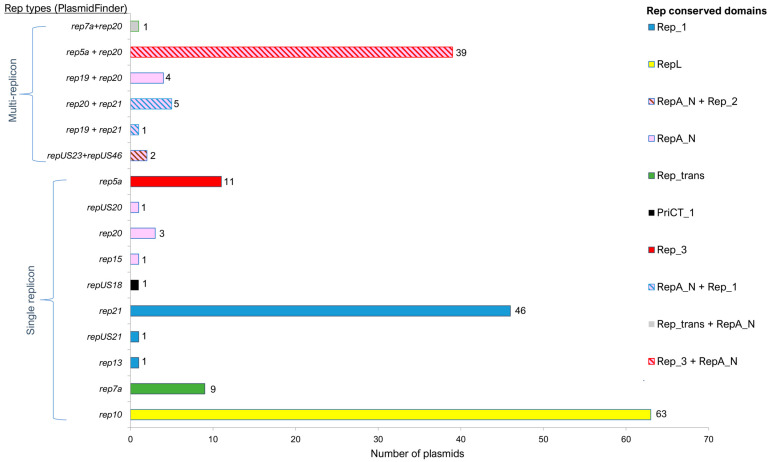
Distribution of replicon types in the plasmids identified from the sequenced MRSA isolates from Malaysia. The *rep* types were identified by the scheme proposed by Jensen et al. [43] and Lozano et al. [44] and available in PlasmidFinder (https://cge.food.dtu.dk/services/PlasmidFinder/, accessed on 1 January 2022). The replication initiation proteins (replicases) were also grouped by their conserved domains [42]. Multi-replicon plasmids encode more than one full-length *rep* gene whereas single-replicon plasmids only encode one full-length *rep* gene.

All seven distinct types of replicases that have so far been found in staphylococcal plasmids [42] were identified in the Malaysian MRSA plasmids (with the replicase family proposed by Jensen et al. [43] and expanded by Lozano et al. [44] and found in the PlasmidFinder database presented in parenthesis): RepL (*rep10*), Rep_trans (*rep7a*), Rep_1 (*rep13*, *rep21*, *rep22*, *repUS21*, and *repUS22*), Rep_2 (*repUS46*), Rep_3 (*rep5a*), RepA_N (*rep15*, *rep19*, *rep20*, *repUS20*, and *repUS23*), and PriCT_1 (*repUS18*). As stated above, these replicase types could be found either individually or in combination within the same plasmid. The most common replicase types were RepL (*n* = 63), RepA_N (*n* = 57), and Rep_1 (*n* = 54), whereas Rep_2 and PriCT_1 replicases were represented by two and one plasmids, respectively (Figure 2). Interestingly, all the multi-replicon plasmids found in this study were a combination of a RepA_N replicase and other replicases, the majority of which was Rep_3 (*n* = 39) (Figure 2).

All 63 members of the RepL type were single-replicon small plasmids with sizes ranging from 2.4 to 2.7 kb, and their high prevalence among *S. aureus* had been previously reported [38,44,52]. These plasmids, which belonged to the pSN2 family [53], encode the replicase gene, a hypothetical protein-coding sequence, and an *ermC* gene, which confers resistance to macrolides, lincosamides, and streptogramin B (MLS_B_) antibiotics (Supplementary Figure S1). The sequence variations observed in these plasmids were either in the non-coding sequences or the *ermC* upstream regulatory sequence, which will be discussed in a later section on antimicrobial resistance. The RepL plasmids appeared to have a reasonably broad host range among the Firmicutes [43] as they have been observed with a high prevalence in *S. epidermidis* [54] but with a much lower prevalence among *Enterococcus faecalis* [55]. These plasmids lack mobilization genes but a lateral transfer of these *ermC* small RepL plasmids between staphylococci and bacteria from different genera had been previously demonstrated. Their transmission was thought to have occurred either by transformation or transduction [56,57,58]. However, mobilization of these RepL plasmids by a relaxase in *trans* mechanism has not been ruled out. One of these transfer mechanisms could perhaps explain the predominance of these plasmids in the Malaysian MRSA isolates.

**Figure 3:**
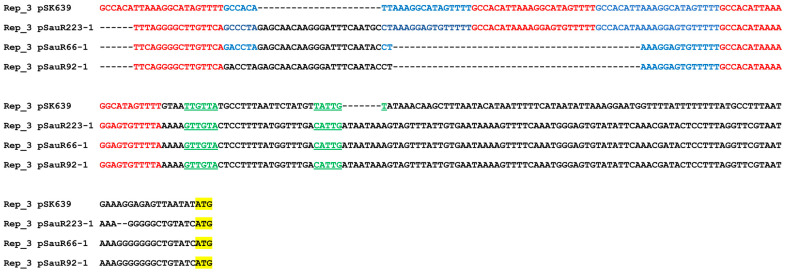
Multiple sequence alignment of the upstream regions of *rep_3* of pSauR223-1, pSauR66-1, and pSauR92-1 (as representative) with the upstream region of *rep_3* of pSK639 as reference (NC_005566.1; [59]). In pSK639, five direct repeats (indicated here in alternating red and blue fonts) are the Rep binding sites that precede the *rep_3* gene. The predicted rep promoter (green and underlined) is found adjacent to these Rep binding sites. The ATG start codon of *rep_3* replicase is highlighted in yellow. Note that in pSauR223-1, pSauR66-1, and pSauR92-1, some of the direct repeat sequences were interrupted or deleted.

Another predominant replicase type in the Malaysian MRSA plasmids was the RepA_N conserved domain (*n* = 57). Only five of the RepA_N plasmids were single-replicon plasmids, while the remaining 52 plasmids were in combination with other Rep types, predominantly with the Rep_3 conserved domain (*n* = 39), and, to a lesser extent, Rep_1 (*n* = 6) (Figure 2). These RepA_N + Rep_3 plasmids, which ranged from 35 to 36 kb in size, were the third-most common plasmid types found in the Malaysian MRSA isolates and encode genes that mediate resistance to the heavy metals arsenate (*arsBC*), cadmium (*cadAC*), and copper (*mco* and *copB*) (Supplementary Figure S1). Four of the RepA_N plasmids appeared to contain two full-length RepA_N replicases (typed as *rep19* and *rep20* by PlasmidFinder). Replicases with the RepA_N domain are usually associated with large plasmids, whereas Rep_1 domain replicases are associated with smaller (<10 kb) plasmids. Replicases with the Rep_3 conserved domain were first reported in a small *S. epidermidis* plasmid pSK639 [59] but later were reported in larger *S. aureus* plasmids [42,60]. Shearer et al. [61] reported that 11 of the 54 RepA_N conserved domain plasmids they investigated also carried a Rep_3 replicase or its truncated remnant, which was suggestive of replicon fusion. This finding was similar to our observation in which the 39 heavy-metal resistance plasmids were found to encode both full-length RepA_N (*rep20*) and Rep_3 (*rep5a*) replicases (Supplementary Table S1). All 39 MRSA isolates harboring this plasmid were of the CC22 lineage, which was the most predominant lineage in the HSNZ isolates (72.2%; *n* = 57/79; Supplementary Table S1), suggesting that these were possibly closely related isolates that might have persisted in the hospital environment. Comparative sequence analysis of the regions found upstream of the replicase genes with related plasmids indicates that the RepA_N replicase is more likely to be functional than the Rep_3 replicase. The divergently transcribed *repA_N-par* (DUF536 protein) region is similar in organization to what has been previously observed in pSK1-family plasmids [23], and the essential genetic elements appear intact. On the other hand, the region found upstream of the Rep_3-encoding replicase gene appears to have deletions in the promoter/*oriV* region [59], which would likely impact the replication function (Figure 3). These RepA_N heavy-metal resistance plasmids shared 100% sequence identity with *S. aureus* plasmid SAP078A (35,508 bp, accession no. GQ900430.1), a heavy-metal resistance plasmid that was previously detected in three MRSA isolates collected from human carriers [62]. When comparing the Malaysian MRSA plasmids such as pSauR66-1 (35,337 bp) with the SAP078A plasmid (35,508 bp), differences were found within a region that encode a hypothetical protein, SAP078A_043 (accession no. ACZ68968.1), namely a 182 bp deletion from nts. 1–182 and a 171 bp deletion from nts. 1144–1283 of the SAP078A plasmid. These deletions were also observed in the other 35–36 kb plasmids from the Malaysian MRSA isolates, with the exception of pSauR133-1 and pSauR192-1, where a larger 443 bp deletion was found from nts. 1–443 of the SAP078A plasmid. Nevertheless, this deletion is still within the SAP078A_043 hypothetical protein-coding sequence. These plasmids also harbor a mobilization gene, *mob/pre* of the MOBV family (that was likely acquired by rolling circle plasmid cointegration followed by deletion), which could facilitate their transmissibility.

Plasmids with the Rep_1 conserved domain were the third-most prevalent plasmid type among the Malaysian MRSA isolates, being found in 54 plasmids, six of these were in combination with a RepA_N replicase and were therefore likely inactive for replication. The high prevalence of Rep_1 type plasmids in MRSA has been described [63], while the co-occurrence of Rep_1 with RepA_N replicases in a single plasmid has also been reported [64]. Rep_1 plasmids, along with those of Rep_trans and RepL types, are usually found in small (<10 kb) staphylococcal plasmids utilizing the rolling circle replication mechanism [42]. In the Malaysian MRSA isolates, 45 of the Rep_1 plasmids were ca. 3 kb cryptic plasmids, which encode only the Rep_1 replicase and a hypothetical protein (of 193 amino acids) (Supplementary Figure S1). This small cryptic plasmid was the second-most predominant plasmid type in our sequenced MRSA isolates, being found in both CC1 and CC22 lineages. It has been shown that some Rep_1 replication initiators can also act as a mobilization relaxase and were designated replicative relaxases [65,66]. The mobilization by replicative relaxases was described by the Grossman laboratory, which also found that integrative and conjugative elements carried by *Bacillus subtilis* efficiently mediate the transfer of three different Rep_1 rolling circle replication plasmids via a mob-independent mechanism [65]. This indicates that replicative-relaxase genes could play a key role in plasmid dissemination when they encounter a conjugative element. Interestingly, these small replicative-relaxase Rep_1 plasmids also carry pWBG749-OT49 *oriT* mimic sequences, except for pZS_Z30-2. pWBG749 is a known large conjugative plasmid in staphylococci, and pWBG749 *oriT* mimic sequences were previously detected on large *S. aureus* antimicrobial-resistance plasmids, such as pIB485, pMW2, and pUSA300HOUMR, which enabled their transmission through a relaxase in *trans* mechanism [26]. The high prevalence of these small Rep_1 cryptic plasmids has been reported previously, with an early study showing that 28 of 30 *S. aureus* clinical isolates were found to harbor a 3.1 kb cryptic plasmid [67]. Later studies also documented a high carriage of similar-sized cryptic plasmids in *S. aureus* [68,69]. Such high occurrences of the same type of cryptic plasmid in *S. aureus* are also highly suggestive of their transmissibility. Rep_1-type plasmids are also frequently found integrated into larger RepA_N and Rep_3 plasmids to form mosaic staphylococcal plasmids [42,60]. The capture of smaller plasmids often results in the accumulation of several resistance genes, resulting in larger multi-resistance plasmids. 

The MRSA isolates sequenced in this study were grouped into CC22 (*n* = 57), CC1 (*n* = 11), CC5 (*n* = 1), and CC8 (*n* = 1) lineages, while nine isolates were with undetermined clonal complexes. As for the database isolates, they were CC8 (*n* = 9), CC1 (*n* = 2), CC5 (*n* = 1), CC30 (*n* = 1), and two isolates with undetermined clonal complexes (Supplementary Table S1). An association of replicons with specific *S. aureus* clonal complex lineages was investigated. The RepA_N replicase was found in all clonal complex lineages; Rep_1 was found in CC1, CC22, CC30, and CC8 lineages; Rep_3 and Rep_L replicons were observed in MRSA isolates belonging to lineages CC1 and CC22; Rep_trans plasmids were identified in CC8 and CC22 isolates; and, finally, the rare PriCT_1 and Rep_2 replicons were found in CC1 and CC5, respectively. Besides the 35–36 kb RepA_N + Rep_3 heavy-metal resistance plasmids being associated with just the CC22 lineage, the other prevalent plasmids (such as the ca. 2.5 kb *ermC*-encoded RepL plasmid and the ca. 3.0 kb cryptic Rep_1 plasmid) were found in several lineages indicating the likelihood of their horizontal transfer. Plasmids with the PriCT_1 and Rep_2 replicases were too few (one each) to be able to make any conclusions regarding their associations with specific clonal complexes.

### 2.3. Conjugative Plasmids, MOB-Typing, and Mobility Potential of the Malaysian MRSA Plasmids

**Figure 4:**
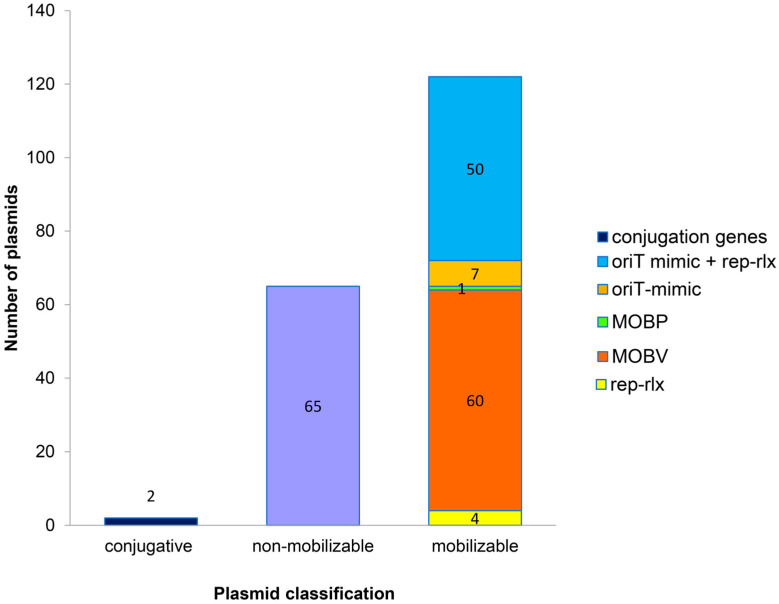
Distribution of the Malaysian MRSA plasmids based on their mobility potential. Note that only two of the identified plasmids (1%, or 2/189) were possibly conjugative, while 64.5% (122/189) plasmids were potentially mobilizable through the conventional mobilization genes *mob/pre* (of the MOBV family) or *mobCAB* (of the MOBP family), or through carriage of replicases with relaxase potential (*rep-rlx*) and/or *oriT*-mimic sequences (i.e., relaxase in *trans* mechanism). The purple-colored box refers to non-mobilizable plasmids and numbers in the figure refer to the number of plasmids.

Besides replicon classification, staphylococcal plasmids can also be classified according to their potential for horizontal transfer, i.e., self-transmissible (conjugative), mobilizable, and non-mobilizable plasmids [28,29,70]. Staphylococcal plasmids exhibit several mobility mechanisms that may facilitate their dissemination, and these were identified from the sequences of the Malaysian MRSA plasmids as described below and summarized in Figure 4. 

**Figure 5:**
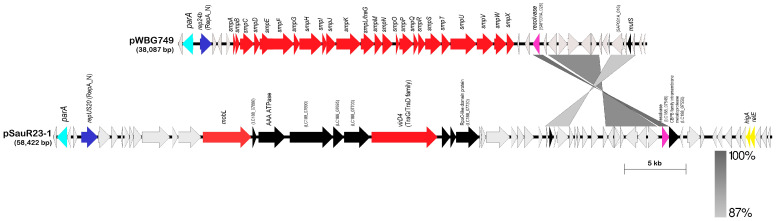
Linear genetic maps of conjugative plasmid pSauR23-1 in comparison with its closest characterized relative, the conjugative plasmid pWBG749 (accession no. GQ900391; [72]). Arrows indicate the size and orientation of known genes and putative ORFs with unknown functions. Regions with greater than 87% nucleotide sequence identity are depicted as shaded grey areas with darker shades of grey indicating higher nucleotide sequence identities (87–100%). Although both plasmids encode replicases with the RepA_N conserved domain (showed as dark blue arrows), the replicases only shared 34% amino acid sequence identity and were typed by PlasmidFinder as *repUS20* for pSauR23-1 and *rep24b* for pWBG749. In both plasmids, a pSK1 *par*-like gene that encodes a single par protein (DUF536) could be found divergently transcribed from their respective *rep* genes (shown as sky-blue arrows and labeled *parA*). The *smpA–smpX* conjugative gene cluster in pWBG749 [26] is indicated in red arrows. Note that no significant sequence identity was observed between the pWBG749 *smp* gene cluster and the putative conjugative gene cluster in pSauR23-1. Two ORFs in pSauR23-1, *mobL* and *virD4* (red arrows), give rise to proteins predicted to be the conjugative relaxase and T4SS coupling protein. Other ORFs are indicated in black arrows with conserved motifs (if any) labeled. Predicted resolvase/serine recombinase genes are shown in pink. The type II toxin-antitoxin system identified in pSauR23-1, *higA-relE*, is colored yellow. Light grey arrows depict hypothetical ORFs.

The occurrence of conjugative plasmids in the Malaysian MRSA isolates was low, with only 1% (2/189) of the plasmids potentially encoding conjugative genes required for DNA processing and mating pair formation. This finding is consistent with previous studies that documented a low prevalence of conjugative plasmids in *S. aureus* [21,29,61,71]. One of these potentially conjugative plasmids, pSauR23-1 (58,442 bp), was found in *S. aureus* SauR23, an MDR MRSA that was isolated from pus in 2016, whereas the other potentially conjugative plasmid, pSAZ10A (35,123 bp), was harbored in MRSA strain SAZ_10, which was also isolated from pus but in 2012 and was sequenced in a previous project (BioProject no. PRJNA503680). The replicase of pSauR23-1 bears the RepA_N domain (typed as *repUS20* by PlasmidFinder) and its replicase gene exhibited 85% identity to the replicase gene of *S. aureus* strain ph1 plasmid pPH1-1 [51] and a lower 34% identity to the RepA_N replicase of pWBG749 [72]. The pSauR23-1 plasmid is possibly conjugative as it encodes a MobL relaxase from nts. 19,570–21,837, a type VI secretion system AAA family ATPase from nts. 22,517–24,376, and a VirD4 superfamily of TraG/TraD-like conjugative transfer coupling protein from nts. 31,588–35,112 (Figure 5). Other coding sequences within and around the region showed no obvious sequence similarities with proteins of known function. The putative conjugative transfer system of pSauR23-1 differs from the system identified in pWBG749, with no sequence identity detected when compared with the pWBG749-encoded *smpA-X* gene cluster [26] or any other conjugation system in the database. However, in the absence of any selectable marker in pSauR23-1, it would be difficult to validate its conjugative potential using conventional conjugation assays. Interestingly, an 8371 bp region within pWBG749 (nts. 8595–16,966 of pWBG749, accession no. GQ900391) shared 95.8% nucleotide sequence identity with pSauR23-1 (nts. 52,322–58,422 and 1–3953, accession no. JAIVEH010000014) but no discernable features could be found within this region, which mainly encodes hypothetical proteins except for a resolvase-like protein of the serine recombinase superfamily (SAP031A_026, nts. 16,424–16,978 of pWBG749) (Figure 5). pSauR23-1 also encodes a type II toxin-antitoxin (TA) system comprising an endoribonuclease toxin of the RelE/ParE family that was paired with an antitoxin of the HigA family. Intriguingly, this is the only plasmid in our collection that harbored a type II TA system. The other plasmids with TA systems harbored only the type I Fst-type TA system, which was identified in 32.8% (62/189) plasmids, and 50 of these plasmids were found to harbor two copies of this TA. The Fst-type I TA has been detected in the chromosome and/or plasmids of a variety of Gram-positive bacteria, with staphylococci being the most common [73,74]. Interestingly, we also detected a putative type III toxin of the ToxN/AbiQ family in two of the MRSA plasmids, i.e., pSauR262 and pSauR269-2 (Supplementary Figure S1). ToxN toxins of this family are similarly endoribonucleases but are usually paired with an RNA antitoxin (*toxI*) made up of direct repeats of 36 nucleotides [75]. However, no such repeats could be found upstream or in the vicinity of the putative *toxN* toxin gene. TA systems have a myriad of proposed functions in bacterial cells, particularly those that are chromosomally encoded [76]. Nevertheless, their ability to mediate the segregational stability of plasmids through the post-segregational killing of any plasmid-free daughter cells was the basis of their initial discovery and they have since been found to stabilize other mobile genetic elements and unstable regions in bacterial chromosomes [77,78,79].

**Figure 6:**
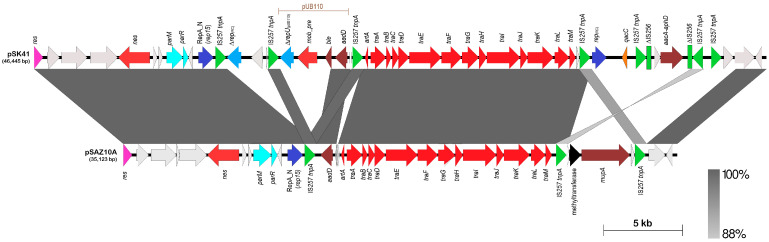
Comparative linear genetic maps of plasmid pSAZ10A with the conjugative plasmid pSK41 (accession no. AF051917; [80]). Arrows indicate the extent and direction of genes and ORFs while regions with >88% nucleotide sequence identities are depicted as shaded grey areas with darker shades of grey indicating higher nucleotide sequence identities. The RepA_N replicase (which was typed as *rep15* by PlasmidFinder) is shown as a dark blue arrow, while lighter blue arrows depict partial or truncated replicase genes, which are labeled with a “Δ” prefix. The *parM-parR* genes that encode the pSK41 plasmid partitioning system are indicated as sky-blue arrows upstream of the *repA_N* replicase gene, while the *res* gene that encodes a serine recombinase, which resolves plasmid multimers into monomers is shown as a pink arrow at the start of the map. The *tra* genes responsible for conjugative transfer are shown as red arrows, as is the relaxase gene, *nes*, that acts on the origin of transfer. Antibiotic resistance genes are indicated in brown arrows while the *qacC* biocide resistance gene in pSK41 is shown in orange. IS element-encoded transposases are depicted as green arrows with the two partial IS*256* elements in pSK41 depicted in green boxes and labeled as ΔIS*256*. In pSK41, IS*257* delimits segments that correspond to cointegrated copies of smaller plasmids, and this includes pUB110, which encodes the *aadD* aminoglycoside and *ble* bleomycin resistance genes (labeled as pUB110) as well as *qacC* on another rolling circle replication plasmid with the replicase labeled as *rep*_(RC)_ [19]. ORFs encoding hypothetical proteins are shown as light grey arrows.

pSAZ10A was similarly a single-replicon conjugative plasmid with a RepA_N domain replicase (typed as *rep15* by PlasmidFinder) and carried an aminoglycoside resistance determinant, *aadD* (*ant(4′)-1b*), and a *mupA* mupirocin resistance gene. However, pSAZ10A belongs to the pSK41 family of conjugative staphylococcal plasmids and shared 99.9% sequence identity over nearly 88% coverage of the 46,445 bp pSK41 (accession no. AF051917) with conservation of the *tra* conjugative transfer region (13 genes from *traA* to *traM*), the *nes*-encoded relaxase, the *res*-encoded recombinase that converts plasmid multimers to monomers, the *parMR* plasmid partitioning system, and the *artA* regulatory gene that coordinates transcription of most of the plasmid’s backbone genes [23,80]. The main differences between pSAZ10A and pSK41 (Figure 6) are (i) the lack of the *qacC/smr* gene and the Tn*4001* hybrid, which contains the *aacA-aphD* aminoglycoside resistance gene in pSK41 [23], with the *mupA* gene in its place and a putative methyltransferase-encoded gene in pSAZ10A; and (ii) the lack of part of plasmid pUB110 and another putative rolling circle plasmid found in pSK41, which are bounded by three copies of the insertion sequence IS*257* [23]. However, the *aadD* aminoglycoside resistance gene is present in pSAZ10A but not the adjacent *ble* bleomycin resistance gene. It should be cautioned that pSAZ10A was assembled from Illumina short reads without any PCR and conventional Sanger sequencing to validate its structure, and the pSK41 family of plasmids contained an abundance of IS*257* and partial IS*256* elements, which would often lead to split assemblies into multiple contigs. Nevertheless, a search for sequences that spanned the missing pSK41 regions in the assembled contigs for *S. aureus* SAZ10A did not lead to any hits inferring their likely absence from the pSAZ10A plasmid. Since the SAZ10A genome sequence was mined from the database, its structure and conjugative potential are unable to be experimentally validated.

On the other hand, 32% (61/189) of the non-conjugative plasmids were classified as mobilizable as they encode mobilization genes (60 plasmids harbored *mob/pre* genes belonging to the MOBV family, while one plasmid contained the *mobCAB* genes belonging to the MOBP family). Further analysis of non-conjugative plasmids that lacked mobilization genes was then performed to investigate if these plasmids have the potential to be mobilized by other known mechanisms (i.e., replicative relaxase and/or carriage of *oriT* mimics, or relaxase in *trans* mechanism). Interestingly, 32% (61/189) of plasmids that lacked conjugation/mobilization genes have the potential to be mobilized by the carriage of *oriT* mimics and/or replicative-relaxase mechanisms. Specifically, seven plasmids contained the pWBG749 family *oriT* mimic sequences, while replication initiators of four Rep_1 plasmids have a relaxase potential, and 50 plasmids possessed both replicative-relaxase as well as pWBG749 family *oriT* mimic sequences. By that, the total number of mobilizable plasmids among our Malaysian MRSA isolates increased to 65% (122/189) (Figure 4 and Supplementary Table S1). This large increase in the number of staphylococcal plasmids with mobilization potential from 32% to 65% is in agreement with previous reports which found that the carriage of *oriT* mimics and replicative relaxases are common but hitherto undetected plasmid mobilization mechanisms in *S. aureus* [21,26,28,29]. Interestingly, 11 of the Malaysian MRSA plasmids were found to carry two *oriT* mimic sequences, namely, the pWBG749 *oriT*-OTUNa and pWBG749 *oriT*-OT49 mimics (Supplementary Figure S1 and Supplementary Table S1). This is not a surprising finding as up to three *oriT* mimic variants have been found on large staphylococcal plasmids such as pWBG744 (27,268 bp) and pWBG762 (54,023 bp), which potentially allows the plasmid to be mobilized by a broader range of conjugative elements [26].

### 2.4. Plasmid-Borne Resistance Genes

**Figure 7:**
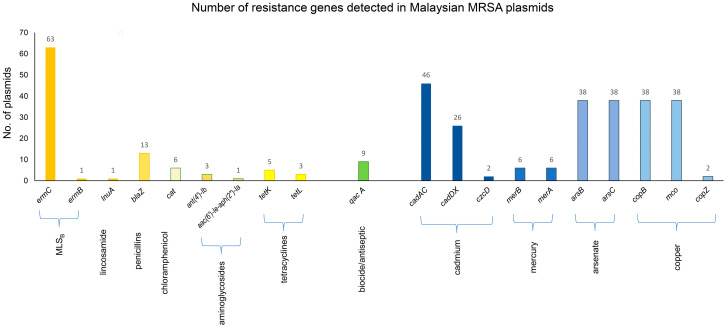
Carriage of antimicrobial, heavy metal, and biocide/antiseptic resistance genes in the Malaysian MRSA plasmids. The category of genes that were detected is categorized as follows: (1) antimicrobial resistance genes (indicated as different shades of yellow to orange bars), i.e., *ermC* and *ermB*, MLS_B_ resistance genes; *blaZ*, penicillin resistance gene; *tetL* and *tetK*, tetracycline resistance genes; *cat*, chloramphenicol resistance gene; *aac(6′)-Ie-aph(2″)-Ia* (also designated *aacA-aphD*) and *ant(4′)-Ib* (also designated *aadD*), aminoglycoside resistance genes; *lnuA*, lincosamide resistance gene; *mupA*, mupirocin resistance gene; (2) biocide/antiseptic resistance gene (indicated as a green bar), i.e., *qacA*, quaternary ammonium compounds resistance gene; (3) heavy metal resistance genes (indicated as different shades of blue bars), i.e., *cadAC*, *cadDX* and *czcD*, cadmium resistance genes; *merA* and *merB*, mercury resistance genes; *arsB* and *arsC*, arsenate resistance genes; and *mco*, *copB* and *copZ*, copper resistance genes.

Sequence analysis revealed that approximately 74% (140/189) of the identified MRSA plasmids encode genes that could confer resistance towards antimicrobial agents, heavy metals, or biocides (Figure 7, Supplementary Figure S1, and Supplementary Table S1). Over half of these resistance plasmids are potentially mobilizable, thereby increasing the likelihood of the spread of these genes to susceptible hosts. Antimicrobial resistance genes were found in 46.6% (88/189) of plasmids with the majority of the resistance plasmids carrying only one resistance determinant (83/88, 94%). The plasmid-encoded antimicrobial resistance genes identified were able to confer their MRSA hosts with reduced susceptibilities towards β-lactams, tetracyclines, aminoglycosides, chloramphenicol, mupirocin, and the macrolides, lincosamides, and streptogramin B (MLS_B_) classes of antimicrobials. Plasmids that hosted antimicrobial resistance gene (s) in *S. aureus* towards different antimicrobial classes were recently reported [81,82].

#### 2.4.1. Inducible versus Constitutive MLS_B_ Resistance

The family of erythromycin ribosomal methylase (*erm*) genes, which confer MLS_B_ resistance was the most prevalent among the Malaysian MRSA isolates (*n* = 64), with *ermC* being found in 63 plasmids (the 2.4–2.7 kb RepL plasmids presented in Section 2.2) and *ermB* only in a single plasmid (pSauR156-1). The *erm* genes mediate MLS_B_ resistance through target site alteration by encoding a 23S ribosomal RNA methyltransferase, which catalyzes the methylation of adenine residues in the peptidyl transferase region of 23S rRNA domain V [83]. The *ermC* gene is commonly seen in staphylococci while the *ermB* gene is more frequently observed in enterococci and streptococci [84,85,86,87,88], which is in agreement with our results. The *erm*-mediated resistant isolates usually exhibit two resistance phenotypes, designated constitutive MLS_B_ (cMLS_B_) and inducible MLS_B_ (iMLS_B_). It is challenging to identify iMLS_B_ phenotype by conventional disk diffusion assays, as isolates with the cMLS_B_ phenotype are resistant to both erythromycin (a macrolide) and clindamycin (a lincosamide), but isolates with the iMLS_B_ phenotype appeared resistant to erythromycin but susceptible to clindamycin. However, iMLS_B_ strains could be induced to clindamycin resistance in the presence of low concentrations of erythromycin and this could only be detected by using the D-test [83,89]. This could lead to therapeutic failure if patients are treated with clindamycin [3,90]. The *erm* genes are usually transcribed on a bicistronic mRNA where the resistance gene is preceded by a regulatory coding sequence for a sensor or leader peptide (sometimes designated *ermL*). In the absence of the inducing antimicrobial, resistance gene expression is attenuated whereas the sensor or leader peptide sequence is constitutively expressed. At low concentrations of the inducing antimicrobial, a fraction of the ribosomes in the cell gets bound with the drug, impeding their progress along the regulatory coding sequence, altering the mRNA conformation, and thereby relieving the attenuation. This, in turn, enables the expression of the resistance gene, leading to a rapid onset of resistance [83,89]. 

**Figure 8:**
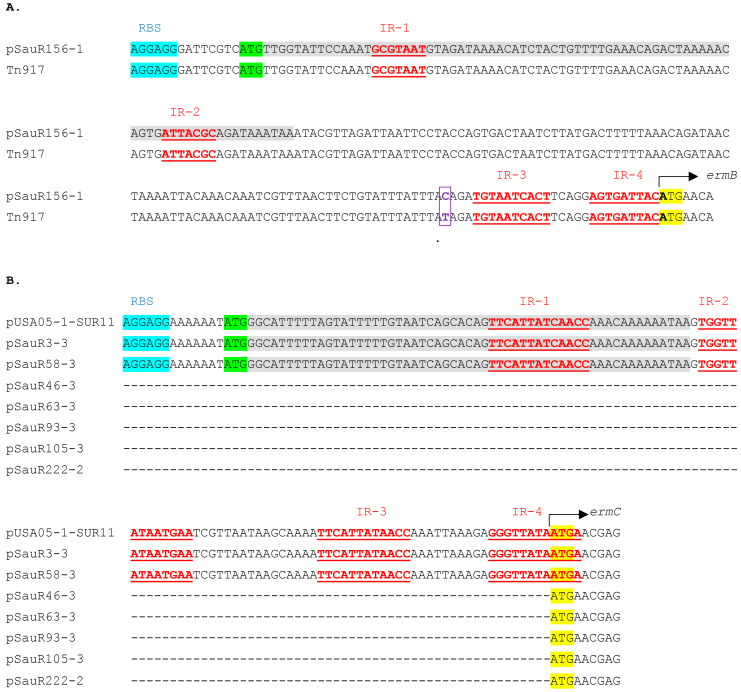
Sequence alignment of *ermB* and *ermC* genes in the Malaysian MRSA plasmids in comparison to the reference plasmids. (**A**) Alignment of the *ermB* leader peptide coding sequence of pSauR156-1 in which the host MRSA strain SauR156 exhibited cMLS_B_ with the *ermB* leader peptide of an inducible *ermB* gene carried on transposon Tn*917*. The T > C mutation in pSauR156-1 upstream of the *ermB* coding sequence is indicated in a purple box. (**B**) Multiple sequence alignment of the *ermC* leader peptide coding region of pSauR3-3 and pUSA05-1-SUR11 (iMLS_B_) and from cMLS_B_ isolates. The start codon of the leader peptide gene is highlighted in green, whereas the start codon of the *erm* gene is highlighted in yellow. Inverted repeats (IR1–IR4) that enabled the regulation by attenuation mechanism for the *erm* gene are underlined with red fonts. RBS, ribosomal binding sites, are highlighted in blue. The leader peptide coding sequence is highlighted in grey. Dashed lines indicate deletions.

In the sequenced Malaysian MRSA isolates, phenotypic testing revealed that the majority of those harboring the plasmid-encoded *ermC* gene showed the iMLS_B_ phenotype (57/63, or 90%), whereas the cMLS_B_ phenotype was observed in the remaining six isolates (namely, SauR46, SauR58, SauR63, SauR93, SauR105, and SauR222). This observation, i.e., the majority of the *ermC*-positive Malaysian MRSA isolates displaying the iMLS_B_ phenotype, is in agreement with other studies [91,92,93]. The single MRSA strain that harbored the *ermB* gene, SauR156, exhibited a cMLS_B_ phenotype. To investigate the likely molecular reasons for the cMLS_B_ phenotype in the six *ermC* plasmid-encoded isolates, a multiple sequence alignment of the regulatory sequences (i.e., leader peptide coding sequence) upstream of the *ermC* structural gene was conducted with known inducible *ermC* region in plasmid pUSA05-1-SUR11 (accession no. NZ_CP014405.1) and pSauR3-3 as a representative *ermC* for the Malaysian iMLS_B_ isolates (Figure 8B). The alignment clearly showed deletion in the *ermC* leader peptide coding sequence in five of these plasmids, i.e., pSauR46-3, pSauR63-3, pSauR93-3, pSauR105-3, and pSauR222-2, which was the likely reason for the cMLS_B_ phenotype observed in their host isolates. Structural modifications including deletions, point mutations, or tandem duplication in the short leader peptide sequence located at the 5′-end of the *erm* gene are known to disrupt the regulatory attenuation mechanism of *erm* expression, leading to the cMLS_B_ resistance phenotype [94,95]. However, the regulatory *ermC* leader peptide coding sequence was present in pSauR58-3 even though its host *S. aureus* SauR58 showed a cMLS_B_ phenotype. Similarly, when aligning the *ermB* sequence in pSauR156-1 with the *ermB* sequence on Tn*917* (accession no. M11180.2) (Figure 8A), the *ermB* leader peptide sequence was clearly present in pSauR156-1 although its host *S. aureus* SauR156 was cMLS_B_. A point mutation (T → C) was observed in pSauR156-1, 28 nts upstream of the *ermB* start codon but it did not affect the leader peptide coding sequence (Figure 8A). A similar point mutation was reported in the *ermB* upstream region of *Streptococcus agalactiae* HM1 and the pneumococcal transposon Tn*1545*, but this mutation did not alter the inducible expression of *ermB* [96]. No deletion or structural variations were thus found in pSauR156-1 and pSauR58 that could explain the cMLS_B_ phenotype of their respective hosts. However, when screening the chromosomal sequences of *S. aureus* SauR58 (accession no. JAINVC000000000) and SauR156 (accession no. JAIUJG000000000) for resistance genes, we found that SauR156 also harbored a *lnuB* gene that encodes for lincosamide nucleotidyl transferase which could confer lincosamide as well as streptogramin B resistance [97]. The combination of the *lnuB* and *ermB* genes in SauR156 would therefore be the possible reason for its cMLS_B_ phenotype, and this was supported by a previous report of cMLS_B_ *S. aureus* isolates that harbored both *lnuB* and *ermB* genes [98]. However, for *S. aureus* SauR58, no similar resistance genes could be found in its chromosome, and without any mutations found in its *ermC* leader peptide or the resistance gene itself, we were unable to provide any compelling reason for its observed cMLS_B_ phenotype.

#### 2.4.2. Other Antimicrobial Resistance Genes

Other antimicrobial resistance genes were found albeit in lower numbers, as shown in Figure 7. Two types of tetracycline resistance genes were identified in the Malaysian MRSA plasmids, i.e., *tetK* (*n* = 5) and *tetL* (*n* = 3). Both these genes encode major facilitator superfamily (MFS) efflux pumps, conferring resistance to tetracycline and, to a lesser extent, doxycycline by an active efflux mechanism [99,100,101]. In a previous study, 69 out of 72 *tetK*-positive *S. aureus* isolates were reported as tetracycline resistant but doxycycline susceptible [102]. In our case, three of the *tetK*-positive isolates (namely, SauR165, SauR223, and ZS-Z37) exhibited tetracycline resistance but doxycycline susceptibility, whereas the remaining five isolates were resistant to both tetracycline and doxycycline. Previous reports indicated that *tetK* was more frequently observed in *S. aureus* whereas *tetL* was uncommon, but both were usually plasmid-borne [103,104,105,106]. 

Six of the Malaysian MRSA isolates (namely, SauR47, SauR84, SauR85, SauR165, SAZ_1, and PPUKM-332-2009) harbored the *cat* gene which confers chloramphenicol resistance, and this was indeed exhibited by four of these isolates (SauR47, SauR84, SauR85, and SauR165). However, SAZ_1 was susceptible to chloramphenicol, whereas the susceptibility phenotype of PPUKM-332-2009 is unknown as it was not stated in the published paper [107]. The *cat* gene was harbored by a Rep_trans small plasmid of 3.7–4.6 kb in all isolates except for SauR165 where the *cat* gene was found in a large (28.6 kb) plasmid, pSauR165-1 (discuss in Section 2.5.1). The reason for the susceptibility of SAZ_1 to chloramphenicol despite the carriage of the *cat* gene is unknown.

The *blaZ* gene, which confers penicillin resistance, was found in 13 plasmids that encode the full *bla* operon consisting of the *blaZ-blaR1-blaI* genes on Tn*552* or ΔTn*552* (which is a variant of Tn*552* that was deleted of the transposase and transposase-related genes), a Tn*7* family transposon that had been proposed to be a likely source of staphylococcal *blaZ* genes [23,32,108]. All isolates that harbored the *bla* operon-encoding plasmids were penicillin-resistant.

Plasmid-encoded aminoglycoside resistance genes were detected in only three MRSA isolates, SauR156, SauR165, and MRSA SAZ_10, with all three isolates harboring the *ant(4′)-Ib* (or *aadD*) gene and SauR165 also containing the *aac(6′)-Ie-aph(2″)-Ia* (or *aacA-aphD*) genes. However, only SauR156 and SauR165 were resistant to amikacin and gentamicin, whereas MRSA SAZ_10 was susceptible to both aminoglycosides. The reason for aminoglycoside susceptibility in MRSA SAZ_10 is unknown as no mutation was detected in the *ant(4′)-Ib* gene it harbored. On the other hand, MRSA SAZ_10 was susceptible to mupirocin despite the carriage of the *mupA* gene, and a closer look at the *mupA* sequence in pSAZ10A (locus tag: FA040_14565) showed the presence of multiple stop codons in the coding sequence. This is due to a frameshift resulting from a single A nucleotide deletion from a row of nine A’s in the wild-type *mupA* gene (using the *mupA/ileS-2* gene from the mupirocin resistant plasmid pPR9 as a comparison; accession no. NC_013653.1; [109]) to only eight A’s in pSAZ10A (nts. 19,415–19,422 in accession no. SWED01000025). 

#### 2.4.3. Heavy Metal and Biocide Resistance Genes

Besides antimicrobial resistance, genes that mediate resistance towards heavy metals such as cadmium, arsenate, mercury, and copper were identified in a total of 66 Malaysian MRSA plasmids (Supplementary Figure S1). Resistance to heavy metals in *S. aureus* and other *Staphylococcus* spp. has been frequently reported [110,111,112]. All 66 heavy metal resistance plasmids in this study harbored cadmium resistance genes with the majority being *cadAC* (*n* = 46) and *cadDX* (*n* = 26), while only two plasmids encoded the *czcD* gene (Figure 7), which has also been implicated in resistance towards cobalt and zinc ions [113]. The *cadA* gene, which encodes an ATPase transporter, can confer resistance to zinc and lead [110,114,115], but such a feature was not observed for *cadD*, which only conferred resistance to cadmium [116]. The mechanism for *cadD* resistance is less well defined, and it was found that *cadD* is related to *cadB*, which confers resistance not through cation efflux but possibly by protecting the cell through binding cadmium in the cell membrane [116,117]. Copper resistance genes were found in 40 plasmids with the majority being the *copB* (*n* = 38) and *mco* (*n* = 38) genes, whereas the *copZ* gene was identified in only two plasmids. Arsenate resistance is the second most abundant heavy-metal resistance determinant, being discovered in 38 plasmids with all of them harboring the *arsB* gene that encodes an arsenate antiporter and the *arsC* gene, which encodes an arsenate reductase. The mercury resistance genes, *merA* and *merB*, were found in only six plasmids, respectively. Nine plasmids were also identified harboring the *qacA* gene that mediates resistance to biocides and antiseptics. The heavy metal and/or biocide resistance genes identified in this study were mainly observed on plasmids that were >14 kb in size, whereas small plasmids generally lack these genes, as has been previously reported [118]. Interestingly, with the exception of pSauR262 and pSauR269-2, all these heavy metals and/or biocide resistance plasmids are classified as mobilizable, thus potentially enabling their spread through horizontal gene transfer.

### 2.5. Other Unique Plasmids and Plasmids of Interest

#### 2.5.1. The Multidrug Resistant pSauR165-1 Plasmid

*S. aureus* SauR165 was isolated from blood in 2017; it displayed resistance to seven antimicrobial classes and harbored three plasmids, pSauR165-1, pSauR165-2, and pSauR165-3 (Supplementary Table S1). Plasmids pSauR165-2 and pSauR165-3 are the small prevalent plasmids found in the Malaysian MRSA isolates previously described, with pSauR165-2 being the 3 kb cryptic Rep_1 plasmid, whereas pSauR165-3 is the 2.5 kb *ermC*-encoding RepL plasmid. On the other hand, pSauR165-1 is 28.6 kb with a RepA_N replicase and encodes resistance to tetracycline (*tetK*), chloramphenicol (*cat*), aminoglycosides [*aadD (ant(4″)-Ib*), *aacA-aphD* (*aac(6′)-Ie-aph(2″)-Ia*)], and lincosamides (*lnuA*), besides also harboring resistance genes for quaternary ammonium compounds (*qacA*) and cadmium (*cadDX*). The pSauR165-1-encoded resistance genes corresponded to the resistance phenotype displayed by its host *S. aureus* SauR165, i.e., resistant to tetracycline, chloramphenicol, amikacin, and gentamicin (all encoded by pSauR165-1) and was iMLS_B_ (pSauR165-3).

**Figure 9:**
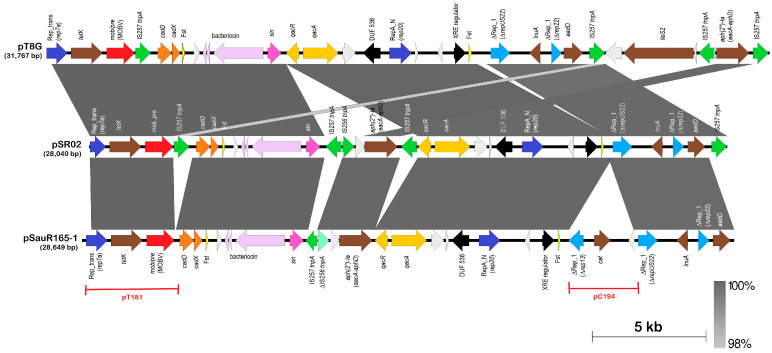
Comparative linear maps of the multidrug-resistant pSauR165-1 plasmid with its close relatives pSR02 plasmid from MRSA strain SR153 (accession no. CP048645; [119]) and pT8G from *Staphylococcus lugdunensis* strain Tlug8G-4 (accession no. KU882684.1; [120]). Arrows indicate the extent and direction of genes and open reading frames (ORFs). Antibiotic resistance genes are shown as brown-colored arrows, cadmium resistance genes (*cadDX*) in orange, and biocide/antiseptic resistance genes (*qacAR*) in gold. Full-length replication initiation (replicase) genes are depicted as dark blue arrows with partial or truncated *rep* genes labeled with the prefix “Δ” and shown in lighter blue. Putative bacteriocin biosynthetic gene clusters are shown in light purple, whereas pink arrows are coding sequences for serine-recombinases, and red arrows are the *mob/pre*-mobilization genes. Insertion sequence (IS)-encoded transposases are depicted as green arrows, light yellow triangles indicate genes for the Fst-type I toxins, black arrows are ORFs with known domains, and ORFs encoding hypothetical proteins are depicted in light grey. Regions of pSauR165-1 that shared sequence identity with smaller plasmids are indicated in red lines labeled with the names of the respective homologous smaller plasmids. Grey-shaded areas show regions with >98% nucleotide sequence identities.

The highest BLASTN hit for pSauR165-1 are plasmids pSR02 from the MRSA strain SR153 (accession no. CP048645) that was isolated in Hangzhou, China, in 2013 [119], and pT8G from the *Staphylococcus lugdunensis* strain Tlug8G-4 (accession no. KU882684.1) that was isolated from Hong Kong at about the same time period [120]. Comparative analysis of these three plasmids (Figure 9) showed that pSauR165-1 harbored an additional *cat* gene that confers resistance to chloramphenicol, and upstream of this gene is a partial Rep_1 replicase gene which is typed as *rep13* by PlasmidFinder. Interestingly, this 2751 bp region, which spans nts. 11,469–14,220 of pSauR165-1 (numbered according to accession no. JAHMGZ010000022.1), showed 99% sequence identity with the small Rep_1 plasmid pC194 (accession no. V01277.1; [121]), covering nts. 162–2910 of the 2910 bp plasmid, which is the prototype for the Rep_1 staphylococcal plasmids [23]. Primers used to cover the gaps in the sequence assembly of pSauR165-1 (Supplementary Table S2) validated this region as part of the pSauR165-1 plasmid, thus inferring that a past integration event with a pC194-type plasmid had occurred, resulting in the current pSauR165-1. However, this is not the only plasmid integration or fusion event that could be detected from the sequences of pSauR165-1. A 3,829 bp region that spanned nts. 18,932–22,762 of pSauR165-1 shared 99% sequence identity with the 4439 bp Rep_trans prototype plasmid, pT181 (accession no. J01764.1) [122]. This 3829 bp region included the full-length Rep_trans replicase gene (*rep7a*), the *tetK* tetracycline resistance gene, and the *mob/pre*-mobilization gene, and was similarly found in plasmids pSR02 and pT8G (Figure 9), suggesting that this pT181-type plasmid integration likely occurred much earlier than the pC194-type integration that was only found in pSauR165-1. Besides these, there were two other partial or truncated Rep_1 replicase genes in pSauR165-1, pSR02, and pT8G that likewise hinted at past plasmid fusion events. In both cases, the partial Rep_1 replicase gene is followed by an antimicrobial resistance gene (*lnuA* for lincosamide resistance and *aadD* for aminoglycoside resistance) (Figure 9). The full-length RepA_N replicase in pSauR165-1, pSR02, and pT8G is possibly the core plasmid sequence as this type of replicase is frequently used by large, theta-replicating staphylococcal plasmids [42]. In contrast, replicases with the Rep_1 and Rep_trans domains are usually used by small, rolling circle replicating plasmids [42], which is likely the reason why the Rep_1 replicases found in pSauR165-1 were all partial or truncated as they were no longer needed for plasmid replication. In fact, if the rolling circle replication functions remain intact then theta replication would be hindered. Hence, it is not a case of redundancy but instead becomes a destabilizing factor for theta replication of the cointegrant plasmid. Inactivation of the functions of rolling circle replication is thus usually necessary in order to maintain the fitness of the co-integrant plasmid [23,42]. 

A 5 kb region that spanned the *qacR-qacA* biocide resistance genes to the RepA_N (*rep20*) replicase gene (nts. 2930–8448 of pSauR165-1) could be repeatedly found in several other large RepA_N (specifically the *rep20* replicase) plasmids identified in this study (namely, pSauR76-1, pZS-Z37-1, pSAZ_1-1, pSAZ10B, pZS-Z30-1, pPUKM-775-2009-1, pPUKM-261-2009-1, and pPUKM-332-2009-1) (Supplementary Figure S1), underlining the modular or mosaic nature of the staphylococcal plasmids which have been well-documented [23,123]. Integration of a pT181-type plasmid with *tetK* was also reported in the ca. 42 kb RepA_N non-conjugative plasmid pWBG715 that was isolated from patients with community-acquired MRSA in Western Australia in the 1990s [124]. Studies on mupirocin-resistant *S. lugdunensis* isolates had shown that pT8G and other related plasmids that were identified from *S. lugdenensis* isolates were likewise mosaic with several modules that were shared among each other and with regions of plasmids pSK41 and pSP01 [120]. However, the authors did not note the presence of the nearly 3.9 kb fragment of the *tetK*-containing pT181 plasmid in some of their collections of *S. lugdenensis* plasmids including pT8G. 

**Figure 10:**
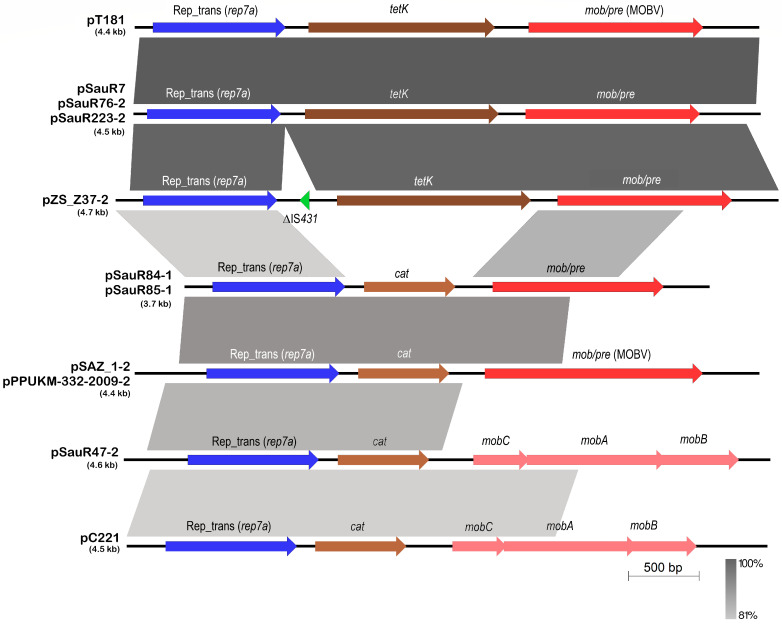
Comparative linear maps of the single-replicon Rep_trans plasmids identified in this study with the reference Rep_trans plasmids pT181 (accession no. NC_001393) and pC221 (accession no. X02166) of the pT181 family. Arrows show the extent and direction of genes and ORFs while regions with >81% nucleotide sequence identities are depicted as shaded grey areas with darker shades of grey indicating higher nucleotide sequence identities. The Rep_trans replicase genes are indicated as dark blue arrows while the *tetK* tetracycline resistance and the *cat* chloramphenicol resistance genes are depicted as brown arrows. Mobilization genes *mob/pre* of the MOBV family, and *mobCAB* of the MOBP family, are shown as red arrows. A truncated version of IS*431* is found in pZS_Z37-2, and this is shown as a green arrow and labeled “ΔIS*431*”.

There were altogether nine single-replicon, small plasmids (<5 kb) of the pT181 family of Rep_trans plasmids in our collection of sequenced Malaysian MRSA isolates (Figure 10). pSauR165-1 was the sole RepA_N + Rep_trans plasmid that was identified. All the single-replicon Rep_trans plasmids encode either the *tetK* tetracycline resistance or the *cat* chloramphenicol resistance genes, and this has been previously noted [42]. The plasmids also contained mobilization systems, either the multigene *mobCAB* system in pC221 and similar plasmids (such as pSauR47-2) or *mob/pre* of the MOBV family in pT181 and its related plasmids. The mosaic nature of this family of plasmids is indeed apparent with discrete functional gene cassettes encoding replication, resistance, and mobilization functions (Figure 10) [23]. 

#### 2.5.2. The pMW2 Family of Resistance Plasmids

**Figure 11:**
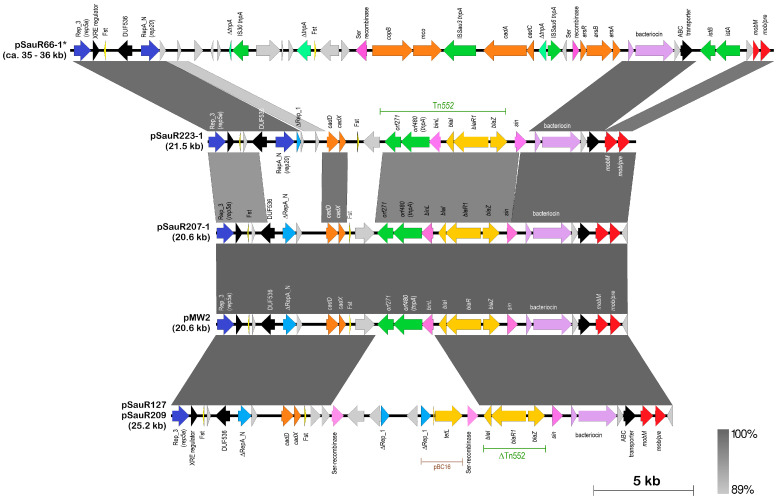
Comparative linear maps of Rep_3 conserved domain plasmids identified in the Malaysian MRSA isolates with the Rep_3 reference plasmid pMW2 (accession no. NC_005011; [126]). Arrows indicate the extent and direction of genes and ORFs with full-length replicase genes colored dark blue and partial or truncated replicase genes in lighter blue and labeled with the prefix “Δ”. Antibiotic resistance genes are shown as gold-colored arrows, while heavy metal resistance genes are in orange. Transposon Tn*552*, which comprises the penicillin-resistant *bla* operon, the *binL* resolvase/recombinase, and the two ORFs that make up the transposase (*orf480* and *orf271*), is indicated. A truncated version of the transposon, which is deleted of *binL* and the two transposase genes, is found in pSauR127 and pSauR209 and labeled as ΔTn*552*. Both pSauR127 and pSauR209 also contained a 2105 bp region next to ΔTn*552* that shared 99.9% nucleotide sequence identity with pBC16 (accession no. U32369.1) from *Bacillus cereus* and is labeled as such in the diagram. This region contained the *tetL* tetracycline resistance gene and part of the Rep_1 replicase of pBC16. Recombinases are shown as pink arrows, mobilization genes as red arrows, transposases are in green, yellow triangles are the Fst toxin of the type I toxin-antitoxin system, light purple arrows are putative bacteriocin-related genes, ORFs with known domains are shown as black arrows, and ORFs encoding hypothetical proteins are indicated as light grey arrows. Regions with >89% sequence identities are depicted as shaded grey areas with darker shades of grey indicating higher nucleotide sequence identities. Plasmid pSauR66-1 (labeled as pSauR66-1*, with an asterisk) was chosen as a representative of the ca. 35–36 kb Rep_3 + RepA_N plasmids encoding several heavy metal resistance genes, and which were found in 39 MRSA isolates in this study.

Plasmid pMW2 (20,654 bp) was first isolated from a community-acquired MRSA strain MW2 that was obtained in North Dakota, USA, in 1998 and contained the 6545 bp β-lactamase transposon Tn*552* (which encoded the *blaZ-blaR1-blaI* operon as well as the *binL* serine resolvase gene and the two transposase genes designated *orf480* and *orf271*) [125,126]. pMW2 has a full-length Rep_3 replicase gene and remnants of a RepA_N replicase gene [23]. A 20,652 bp plasmid (pSauR207-1) that was 99.9% identical in sequence to pMW2 was isolated from *S. aureus* SauR207, a MDR MRSA that was obtained from blood in 2019 and which also harbored one other smaller plasmid, the 2.5 kb *ermC*-encoding pSN2 family (RepL) plasmid (pSauR207-2) (Supplementary Table S1). Like pMW2, pSauR207-1 harbored Tn*552*, the cadmium resistance *cadDX* genes, putative genes for bacteriocin synthesis, and putative mobilization genes (Figure 11). 

Two derivatives of pMW2/pSauR207-1 were found in the plasmid collection of this study. In one of these derivatives, pSauR127/pSauR209, a truncated 3514 bp version of Tn*552* was present, spanning only the *blaZ-blaR1-blaI* operon and bereft of the transposition-related genes. Two partial Rep_1 replicase genes and a *tetL* tetracycline resistance gene were found in a 7360 bp fragment that was bounded by two serine recombinase-like genes, and which were absent in pMW2/pSauR207-1 (Figure 11). One of these serine recombinase genes was adjacent to *blaI* and replaced the *binL* recombinase of Tn*552*, albeit in the opposite orientation. It appears this 7360 bp region is a patchwork of at least two smaller Rep_1 plasmids, one of which encoded *tetL*. A BLASTN search found a 2105 bp region that spanned the partial Rep_1 replicase and the *tetL* genes sharing 99.9% sequence identity with a 4630 bp plasmid pBC16 from *Bacillus cereus* (accession no. U32369.1). 

The other pMW2 derivative, pSauR223-1, differed from pMW2/pSauR207-1 in that it encoded a full-length RepA_N replicase (typed as *rep20* by PlasmidFinder) and possibly an inactive Rep_3 (*rep5a*) replicase. The RepA_N replicase of pSauR223-1 is 315 aa in length whereas the partial RepA_N replicase coding sequence in pMW2/pSauR207-1 was 225 aa and had several internal stop codons due to frameshifts in the coding sequence. The RepA_N replicase from pSauR223-1 only shared 35% sequence identity with the partial RepA_N replicase from pMW2/pSauR207-1. Downstream from the RepA_N replicase coding sequence in pSauR223-1, a partial Rep_1 replicase could be found (Figure 11). The full-length Tn*552* as well as the *cadDX* genes were present in pSauR223-1. 

Comparative analysis also showed that the ca. 35–36 kb RepA_N + Rep_3 heavy-metal resistance plasmid (exemplified by pSauR66-1 in Figure 11) that was prevalent in the Malaysian MRSA isolates (see Section 2.2) was related to pMW2. Like pSauR223-1, pSauR66-1 had a full-length RepA_N replicase, and their translated amino acid sequences were 100% identical. The predicted partitioning system (*par*) of RepA_N replicases is divergently transcribed from the *repA_N* gene and is usually annotated as DUF536 [124]. The translated amino acid sequences for this coding sequence are likewise 100% identical between the pSauR223-1 and pSauR66-1 plasmids. Interestingly, although pMW2, pSauR207-1, and pSauR127/pSauR209 had partial RepA_N-encoded genes, these plasmids had a full length, divergently transcribed DUF536-encoded reading frames. However, this putative *par* gene of pMW2 only shared 40% amino acid sequence identity with the homologs from pSauR66-1 and pSauR223-1. Since the partial *repA_N* from pMW2 and its closely related plasmids only shared 35% sequence identity with the *repA_N* from pSauR66-1 and pSauR223-1, this inferred that the DUF536/*par*-*repA_N* module in pMW2 and its closely related variants is different from that found in pSauR66-1 and pSauR223-1. Nevertheless, all these plasmids (i.e., pSauR66-1 and pSauR223-1, pMW2 and its closely related variants) shared a common Rep_3 replicase gene and a downstream *xre* gene (helix-turn-helix regulator) that were 100% identical in their amino acid sequences. However, the *rep_3* upstream sequences, which comprised the five direct repeats of the Rep_3 binding sites along with the predicted *rep_3* promoter of these plasmids, were altered with deletions and insertions when compared to the reference Rep_3 plasmid pSK639 (see Section 2.2 and Figure 3), indicating that in these plasmids, the Rep_3 replicase is possibly non-functional and, plasmid replication is likely via the RepA_N replicase.

pSauR66-1 also shared the putative bacteriocin synthesis gene cluster as well as the *mob/pre*-mobilization genes with the other plasmids. These genes are contiguous with the *rep_3* and *xre* genes in pSauR223-1 and pMW2 but are interrupted by an IS*21*-like family element (which encode the IS*21*-type *istA* and *istB* transposases but without any sequence identities with known IS elements in the database) in pSauR66-1 (Figure 11). The ca. 10 kb region in pSau223-1 that spanned the *cadDX* genes to the *sin* recombinase downstream of Tn*552* is replaced by a ca. 21.4 kb fragment in pSauR66-1 that contained various heavy metal resistance genes (the copper resistance genes *copB* and *mco*, cadmium resistance *cadCA*, and arsenate resistance *arsR-arsBC* genes) interspersed with various IS elements (full-length IS*Sau3* and IS*Sau6*) and partial IS elements/transposases (Figure 11).

### 2.6. Limitations of the Study

To our knowledge, this is the most comprehensive and detailed analysis of plasmids found in Malaysian MRSA isolates so far. A major limitation of this study is that the majority of the MRSA genome sequences that were analyzed (79/94) were from a single, albeit the main, tertiary public hospital in the state of Terengganu, and thus could not be said to represent the entire nation. Nevertheless, some of the MRSA genomes that were obtained from GenBank (15/94) and analyzed together in this study originated from other states within Malaysia. A more comprehensive survey of MRSA isolates from across Malaysia would, of course, have been ideal to enhance the reliability of the findings but, unfortunately, we were hampered by the scarcity of such genome sequence data that are currently available in the public databases. Another limitation is that this study did not consider environmental factors that may influence the prevalence and characteristics of the plasmids, such as the use of antibiotics. These factors should be explored in further studies to gain a better understanding of the dynamics of plasmid carriage in MRSA isolates. Finally, it should be noted that the MRSA genomes in this study were sequenced using a short-read, 150-bp paired-end sequencing strategy on the Illumina/DNBSEQ platforms. The assembly of such sequence data typically results in multiple contigs due to the presence of repeated sequences and for large plasmids interspersed with several IS elements, obtaining their complete sequence would be a challenge [127,128]. We tried to minimize this by closing the gaps in between contigs through PCR and conventional Sanger sequencing (see Section 3.5) whenever possible, but the validation of the assembled large plasmid sequences would typically require long-read sequencing using either the Pacific Biosciences (PacBio) or Oxford Nanopore Technologies (ONT) platforms, which are higher in cost [127,128]. We have recently done so for one of the MRSA isolates, SauR3, and hybrid assembly of the Illumina and ONT reads validated the presence of three plasmids in the isolate [129].

## 3. Materials and Methods

### 3.1. Bacterial Isolates

A total of 94 MRSA isolates were included in this study as follows: (i) 79 MRSA clinical isolates that were obtained between the years 2016 and 2020 from Hospital Sultanah Nur Zahirah (HSNZ), the main tertiary public hospital in Kuala Terengganu (which is situated in the east coast of Peninsular Malaysia), were sequenced in this study (deposited under the BioProject accession no. PRJNA722830); (ii) assembled contig sequences of 15 Malaysian MRSA isolates that were retrieved from National Center for Biotechnology Information (NCBI) genomes database (https://www.ncbi.nlm.nih.gov/genome, accessed on 1 January 2022). The accession/BioProject numbers of these previously assembled genomes are AOCQ00000000, ANPO00000000, AMRB00000000, AMRC00000000, AMRD00000000, AMRE00000000, and PRJNA503680. Approval for the collection of MRSA isolates from HSNZ was obtained from the Medical Research and Ethics Committee, Ministry of Health Malaysia, under the National Medical Research Registry Protocol No. NMRR-15-2369-28130 (IIR) and NMRR-19-3702-52104 (IIR).

### 3.2. Validation of MRSA Isolates and Determination of Their Antimicrobial Susceptibility Profiles

All MRSA isolates from HSNZ were validated by a combination of phenotypic and molecular methods [3,90]. Isolates were subcultured on mannitol salt agar with presumptive *S. aureus* colonies appearing yellow (i.e., mannitol fermenter). Isolates were also subjected to cefoxitin (30 µg) disk diffusion assay with an inhibition zone of ≤21 mm indicative of MRSA [3]. For molecular confirmation of MRSA, PCR was performed for the detection of the *nucA* and *mecA* genes using established primers [3]. Antimicrobial susceptibility profiles for all isolates were determined by disk diffusion as previously described [3,90] using a panel of 18 classes of antimicrobials encompassing 26 antibiotics and interpreted using breakpoints provided by the Clinical and Laboratory Standards Institute (CLSI) and the European Committee on Antimicrobial Susceptibility Testing (EUCAST) [130,131]. Macrolide-lincosamide-streptogramin B (MLS_B_) resistance phenotypes, i.e., constitutive (cMLS_B_), inducible (iMLS_B_), macrolide-streptogramin B (MS), or susceptible, were determined using the D-test [130].

### 3.3. Genomic DNA Extraction

A 5 mL LB broth was inoculated from a single colony of MRSA grown overnight at 37 °C on mannitol salt agar plate. The inoculated broth was incubated overnight with shaking at 37 °C. Bacterial cells were harvested by centrifugation at 13,000× *g* for three minutes, and then total genomic DNA was extracted for WGS using the Presto™ Mini gDNA Bacteria Kit (Geneaid Biotech Ltd., New Taipei City, Taiwan) according to the manufacturer’s instruction. An amount of 200 μL of lysostaphin (3 µg/mL) (Sigma-Aldrich, Saint Louis, MO, USA) was used to aid in the lysis of staphylococcal cells prior to DNA extraction. The quality and concentration of the extracted genomic DNA were checked using an Implen Nanophotometer^®^ spectrophotometer (Implen, Munich, Germany) as well as electrophoretic separation at 100 V for 45 min using 1% (*w*/*v*) agarose gel in 1× TAE buffer, stained with SYBR™ Safe gel stain (Thermo Fisher Scientific, Waltham, MA, USA), and then visualized under UV light. 

### 3.4. Whole Genome Sequencing, De Novo Assembly, and Assembly Evaluation

The genomes of the selected MRSA isolates were sequenced by commercial sequencing providers with a paired-end sequencing strategy using an Illumina HiSeq platform (HiSeq-PE150) (Novogene, Singapore) or DNBSEQ platform (Beijing Genome Institute, Beijing, China). The cleaned data obtained from the commercial providers were de novo assembled using the SPAdes assembler (v3.13.0) coupled with BayesHammer [132] and Unicycler (v.0.4.8) [133]. The quality of the assembled contigs were evaluated using QUAST (Assembly Quality Assessment Tool for genome assemblies) available at PathoSystems Resource Integration Centre (PATRIC) [134,135]. 

### 3.5. Plasmid Sequence Identification, Replicon Typing and Annotation

Plasmids were identified from whole genome assembled contigs according to a previously proposed protocol [136]. The *rep*-carrying contig (s) and replicon types were identified using PlasmidFinder version 2.1 [45,46] available at the Centre for Genomic Epidemiology (https://cge.food.dtu.dk/services/PlasmidFinder/, accessed on 1 January 2022). For draft plasmid sequences that spanned multiple contigs, gaps in between contigs were amplified by PCR and conventional Sanger sequencing of the resulting amplicons, if possible (Supplementary Table S2 lists primers used in gap closure for the various plasmid contigs in this study). Some of the completed plasmid contigs were also validated by performing PCR using outward-directing primers, whereby a positive amplification of the expected size would confirm the circular (i.e., plasmid) nature of the respective contigs. The final assembled plasmid sequences were annotated using the PATRIC RASTtk-enabled Genome Annotation Service [137]. The identified plasmids were named with a prefix “p” letter followed by the strain code, for example, pSauR7 for the plasmid from *S. aureus* SauR7. When multiple plasmids were detected in a single isolate, they were ordered according to size from the largest to smallest, for example: pSauR47-1, pSauR47-2, pSauR47-3, and so on.

### 3.6. Bioinformatic Analysis of the Identified Plasmids

The identified plasmid sequences were further examined using several bioinformatics tools as follows: MOB typing was determined using the MOBscan web interface [138] that utilized the hmmscan function of the HMMER3 software [139] to search against MOBfamDB, a curated relaxase profile database; the Comprehensive Antibiotic Resistance Database (CARD) [140] and ResFinder [141,142,143] were used to identify antibiotic resistance genes; the BacMet database was used to screen heavy metal and biocide resistance genes [144]; Virulence-Finder was used to identify putative virulence genes [145,146]; the ISfinder database [147] was used to identify insertion sequences (IS) in the assembled contigs; and the T4SS secretion components were determined using SecReT4 [148] and CONJscan module of MacSyFinder [149]. Moreover, *oriT*-mimic sequences identification was carried out using BLASTN local alignment, where *oriT* sequences of known conjugative staphylococcal plasmids such as the pWBG749 *oriT* subfamilies and pSK41/pGO1 *oriT* were retrieved and used as queries [4,26]. Detection of replicative-relaxase-encoded genes was performed by aligning previously known replicative-relaxase protein sequences with the replication initiator amino acid sequences of the target plasmids using MAFFT version 7.48, a multiple alignment program (https://mafft.cbrc.jp/alignment/, accessed on 1 January 2022). Toxin and antitoxin genes were identified by Rasta bacteria [150] and TADB database [151]. Graphical maps of the annotated plasmid genomes were created using SnapGene^®^ 5.1.5 (from Insightful Science; available at snapgene.com, accessed on 1 January 2022) and then further modified. Homology of some of the identified Malaysian MRSA plasmids to existing reference plasmids was carried out using the NCBI BLAST + tool kit (ftp://ftp.ncbi.nlm.nih.gov/blast/executables/blast+/LATEST/, accessed on 1 January 2022) and subsequently visualized using EasyFig 2.1 (https://mjsull.github.io/Easyfig/, accessed on 1 January 2022).

## 4. Conclusions

The characteristics and diversity of plasmids found within MRSA isolates from Malaysia were presented in this study. We found that the majority of the plasmids found in the Malaysian MRSA isolates were small (<5 kb), with the 2.4–2.7 kb, *ermC*-encoding, RepL-type plasmid being predominant (*n* = 63 out of 94 MRSA isolates, or 67%). The carriage of this *ermC* plasmid was also responsible for the iMLS_B_ resistance phenotype observed in most of the Malaysian MRSA isolates, except for several isolates which showed the cMLS_B_ phenotype due to the absence of the *ermC* leader peptide coding sequence in this plasmid. Another small (ca. 3 kb) plasmid of the Rep_1-type, albeit cryptic, was also predominant (*n* = 45), and its prevalence in the absence of any obvious selective advantage to its host could be due to the mobilization potential of the Rep_1 replicase and the presence of *oriT*-mimic sequences. This would, however, require future experimental validation. These “alternative” mobilization mechanisms were found to increase the number of potentially mobilizable plasmids in our collection two-fold. In stark contrast, only two of the 189 identified plasmids were likely conjugative with pSAZ10A (which encoded mupirocin and aminoglycoside resistance genes) being closely related to the conjugative staphylococcal plasmid pSK41, whereas pSauR23-1 is a novel albeit cryptic 58.4 kb plasmid with no known functional homologs to its putative conjugative genes. The two conjugative plasmids identified in this study encode a RepA_N domain replicase, which was the main replicase gene found in the large (>15 kb) MRSA plasmids, either by itself (*n* = 5) or more often in combination with other replicases (*n* = 52). A ca. 35–36 kb RepA_N + Rep_3 plasmid was in fact the third most abundant plasmid type (*n* = 39) in the Malaysian MRSA isolates and harbored several genes that could confer resistance to heavy metals including arsenate, cadmium, and copper. The reason for the widespread occurrence of this relatively large plasmid in the Malaysian MRSA isolates is unknown as no obvious selective advantage could be gleaned from its genetic content. Nevertheless, the finding that this plasmid was only found in CC22 isolates is suggestive of the clonal spread of this specific lineage in the hospital environment. Our sequence analyses also underlined the mosaic nature of staphylococcal plasmids, with several large plasmids having signature sequences of smaller plasmids (especially partial Rep_1-type replicases) strongly suggesting past fusion or cointegration events. These first few snapshots of the plasmidomic landscape in the Malaysian clinical MRSA isolates have given us important insights into the nature of this priority bacterial pathogen circulating in the hospital in Terengganu and to a lesser extent in Malaysia, particularly its horizontal gene pool which plays such an important role in its evolutionary adaptation.

## Data Availability

The genomes of the *S. aureus* isolates sequenced in this study are deposited in GenBank under BioProject accession no. PRJNA722830. The individual accession numbers for each isolate were listed in Supplementary Table S1.

## Supplementary Materials

The following supporting information can be downloaded at https://www.mdpi.com/article/10.3390/antibiotics12040733/s1. Table S1: list of plasmids and their genetic characteristics identified in this study along with their respective GenBank accession numbers. Table S2: list of primers used for gap closures and sequence validation of identified plasmids. Figure S1: linear maps of the plasmid types that were found in the present study.
