# Supplementary material for: The Plasmidomic Landscape of Clinical Methicillin-Resistant Staphylococcus aureus Isolates from Malaysia
Source: Antibiotics (Basel). 2023 Apr 9;12(4):733. doi: 10.3390/antibiotics12040733 (PMC10135026; doi:10.3390/antibiotics12040733)
Supplement: Supplementary file 1 [file antibiotics-12-00733-s001.zip › List of Suppl. Material (Figure S1, Table S1 & Table S2).pdf]

The plasmidomic landscape of clinical methicillin-resistant *Staphylococcus aureus* isolates from Malaysia

Esra'a I. Al-Trad [1], Ching Hoong Chew [2*], Ainal Mardziah Che Hamzah [2], Zarizal Suhaili [3], Nor Iza A. Rahman [1], Salwani Ismail [1], Suat Moi Puah [4], Kek Heng Chua [4], Stephen M. Kwong [5] and Chew Chieng Yeo [1*]

Supplementary materials:

Supplementary Figure

Supplementary Figure S1. Linear maps of the plasmid types that were found in this study. (A) Maps of single replicon plasmids with the RepL, Rep_1, and Rep_trans domains (i.e., plasmids that replicate using the rolling circle replication (RCR) mechanism); (B) single replicon plasmids with the PriCT_1 and Rep_3 conserved domains (plasmids that replicate using the theta replication mode); (C) multi-replicon plasmids. Abbreviations: *rep*, replication initiation (replicase) gene; *par*, plasmid partitioning protein-encoding gene; *nes*, relaxase gene; *tra*/*virD4*, transfer genes; *mobL*/*mob*/*pre*, mobilization/recombination genes; *tetK*/*tetL*, tetracycline resistance genes; *ermC*/*ermB*, MLS_B resistance genes [*ermC(L)* and *ermB(L)* refer to the leader peptide coding sequence that preceded their respective structural genes and is involved in regulation by attenuation (see main text for details)]; *arsC*/*arsB*/*arsR*, arsenic resistance genes; *qacA*/*qacR*, biocide/antiseptic resistance genes; *cadAC*/*cadDX*, cadmium resistance genes; *mco*, *copB*, or *copZ*, copper resistance genes; *merA*/*merB*, mercury resistance genes, *blaZ-blaR1-blaI*, penicillin resistance operon; ANT*(4')-IB* (also designated *aadD*) and *AAC(6')-Ie-APH(2'')-Ia* (also designated *aacA-aphD*), aminoglycoside resistance genes; IS, insertion sequence; *sin*/*bin*: serine recombinase; *mutS*, mismatch DNA repair protein; Fst, toxin of the Fst type I toxin-antitoxin system. Partial or truncated genes or elements are designated with the prefix "Δ". Light grey arrows represent open reading frames (ORFs) for hypothetical proteins; black arrows for ORFs with known domains or functions.

Supplementary tables

Table S1. List of plasmids and their genetic characteristics identified in this study along with their respective GenBank accession numbers.

Table S2. List of primers used for gap closures and sequence validation of identified plasmids.
