# Supplementary material for: The Plasmidomic Landscape of Clinical Methicillin-Resistant Staphylococcus aureus Isolates from Malaysia
Source: Antibiotics (Basel). 2023 Apr 9;12(4):733. doi: 10.3390/antibiotics12040733 (PMC10135026; doi:10.3390/antibiotics12040733)
Supplement: Supplementary file 1 [file antibiotics-12-00733-s001.zip › SUPPL FIG S1 (PDF).pdf]

Supplementary Figure S1

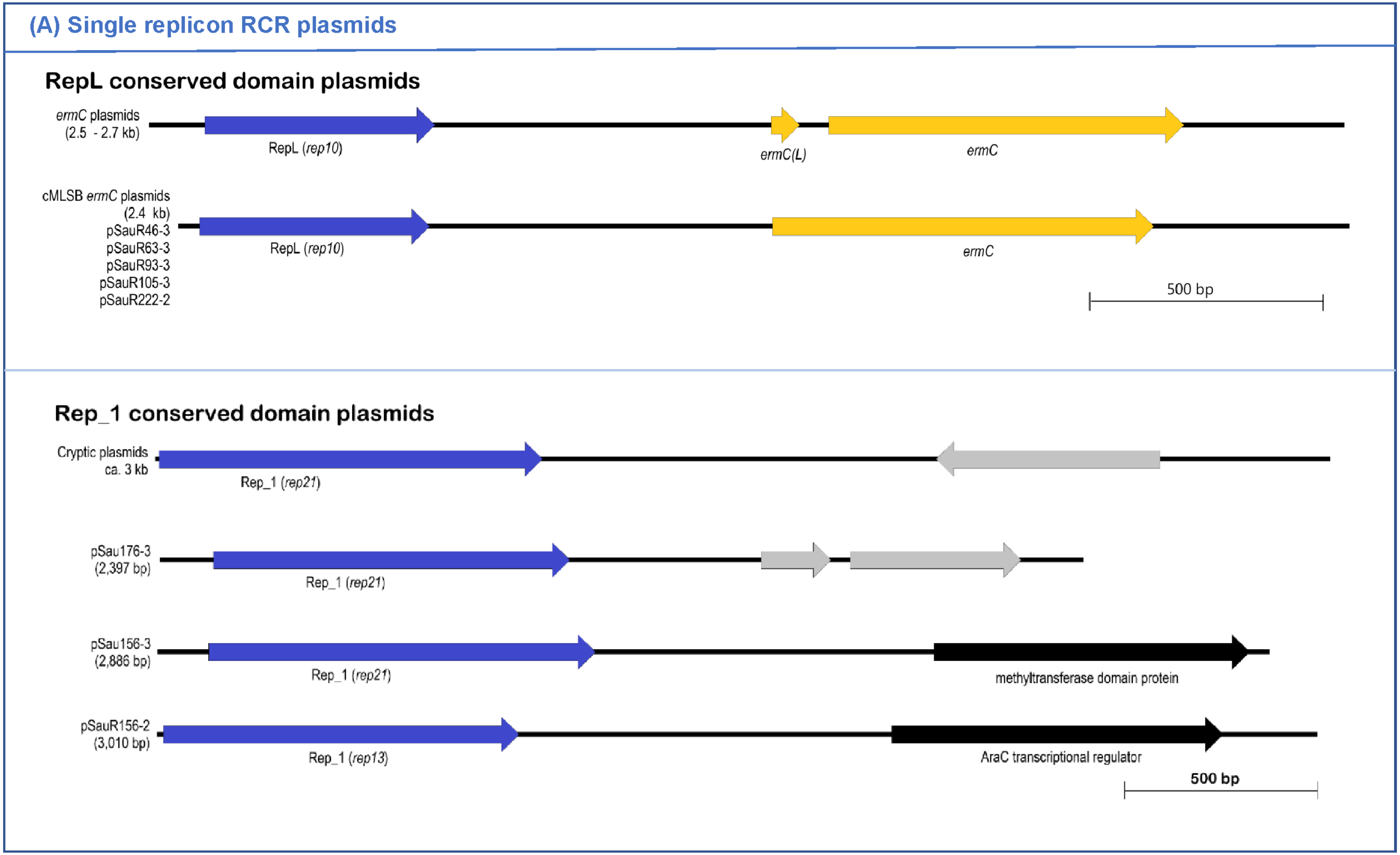

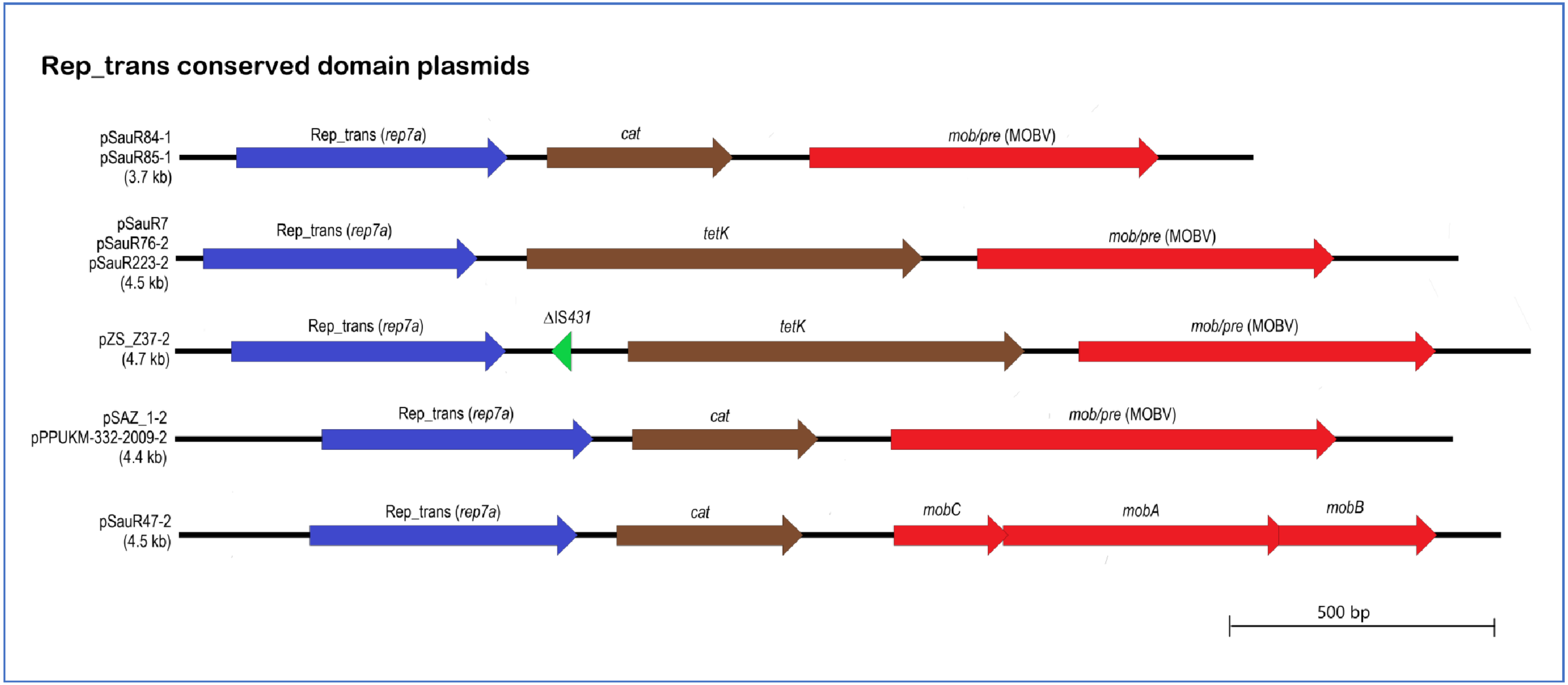
Rep_trans conserved domain plasmids
pSauR84-1
pSauR85-1
(3.7 kb)
Rep_trans (rep7a)
cat
mob/pre (MOBV)
pSauR7
pSauR76-2
pSauR223-2
(4.5 kb)
Rep_trans (rep7a)
tetK
mob/pre (MOBV)
pZS_Z37-2
(4.7 kb)
Rep_trans (rep7a)
ΔIS431
tetK
mob/pre (MOBV)
pSAZ_1-2
pPPUKM-332-2009-2
(4.4 kb)
Rep_trans (rep7a)
cat
mob/pre (MOBV)
pSauR47-2
(4.5 kb)
Rep_trans (rep7a)
cat
mobC
mobA
mobB
500 bp

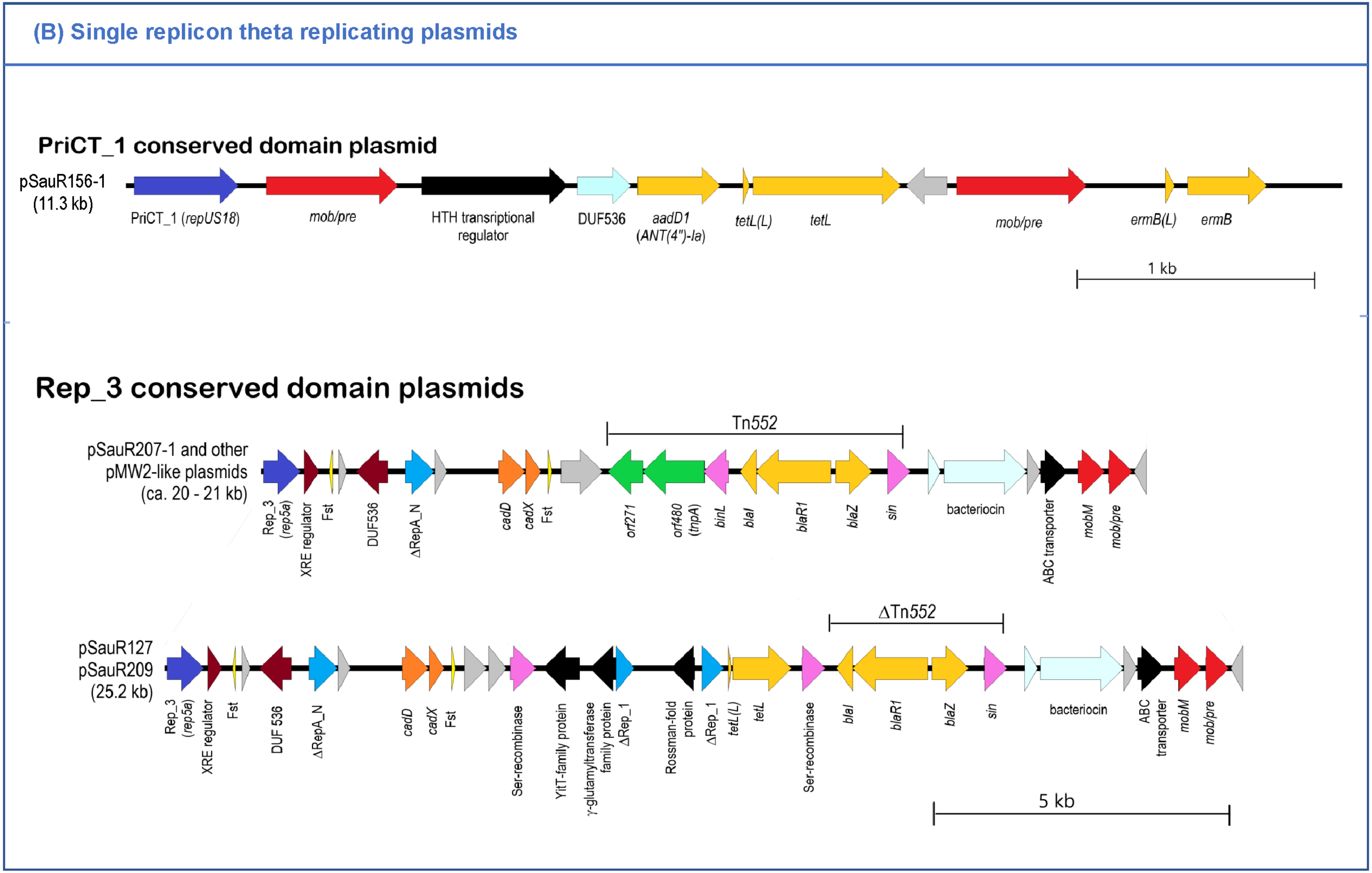
(B) Single replicon theta replicating plasmids
PriCT_1 conserved domain plasmid
pSauR156-1
(11.3 kb)
PriCT_1 (repUS18)
mob/pre
HTH transriptional regulator
DUF536
aadD1
(ANT(4")-Ia)
tetL(L)
tetL
mob/pre
ermB(L)
ermB
1 kb
Rep_3 conserved domain plasmids
Tn552
pSauR207-1 and other
pMW2-like plasmids
(ca. 20 - 21 kb)
Rep_3
(rep5a)
XRE regulator
Fst
DUF536
ΔRepA_N
cadD
cadX
Fst
orf271
orf480
(tnpA)
binL
blaI
blaR1
blaZ
sin
bacteriocin
ABC transporter
mobM
mob/pre
ΔTn552
pSauR127
pSauR209
(25.2 kb)
Rep_3
(rep5a)
XRE regulator
Fst
DUF 536
ΔRepA_N
cadD
cadX
Fst
Ser-recombinase
YitT-family protein
γ-glutamyltransferase family protein
ΔRep_1
Rossman-fold protein
ΔRep_1
tetL(L)
tetL
Ser-recombinase
blaI
blaR1
blaZ
sin
bacteriocin
ABC transporter
mobM
mob/pre
5 kb

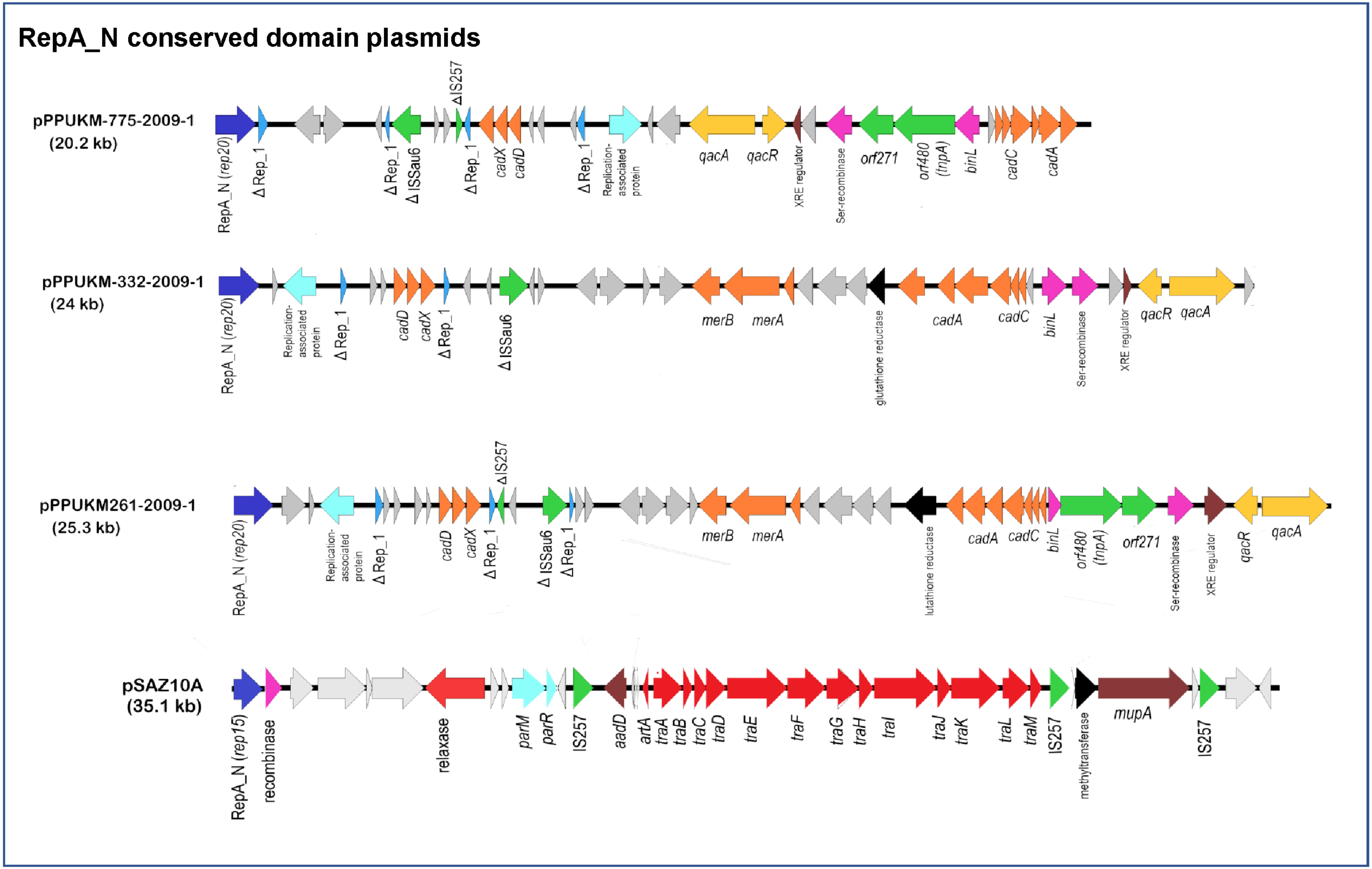

RepA_N conserved domain plasmids
pPPUKM-775-2009-1
(20.2 kb)
RepA_N (rep20)
Δ Rep_1
Δ Rep_1
Δ ISSau6
Δ IS257
Δ Rep_1
cadX
cadD
Δ Rep_1
Replication-associated protein
qacA
qacR
XRE regulator
Ser-recombinase
orf271
orf480 (tnpA)
binL
cadC
cadA
pPPUKM-332-2009-1
(24 kb)
RepA_N (rep20)
Replication-associated protein
Δ Rep_1
cadD
cadX
Δ Rep_1
Δ ISSau6
merB
merA
glutathione reductase
cadA
cadC
binL
Ser-recombinase
XRE regulator
qacR
qacA
pPPUKM261-2009-1
(25.3 kb)
RepA_N (rep20)
Replication-associated protein
Δ Rep_1
cadD
cadX
Δ Rep_1
Δ IS257
Δ ISSau6
Δ Rep_1
merB
merA
lutathione reductase
cadA
cadC
binL
orf480 (tnpA)
orf271
Ser-recombinase
XRE regulator
qacR
qacA
pSAZ10A
(35.1 kb)
RepA_N (rep15)
recombinase
relaxase
parM
parR
IS257
aadD
artA
traA
traB
traC
traD
traE
traF
traG
traH
traI
traJ
traK
traL
traM
IS257
methyltransferase
mupA
IS257

RepA_N conserved domain plasmids (cont'd)

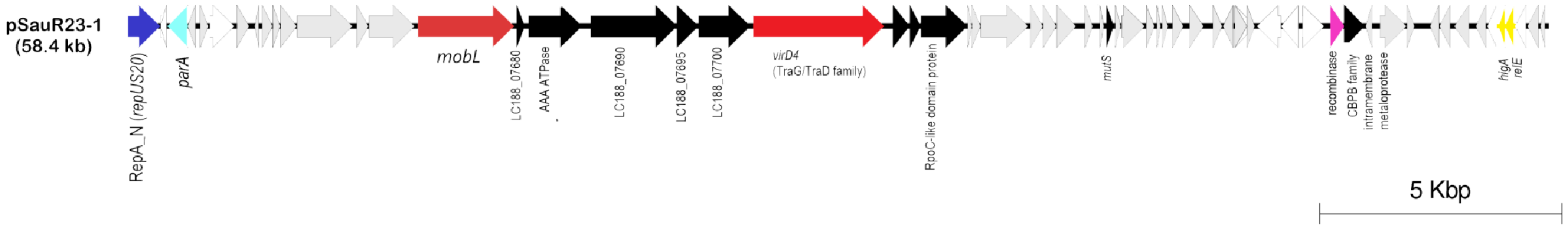

(C) Multi-replicon plasmids

RepA_N + Rep_trans plasmid:

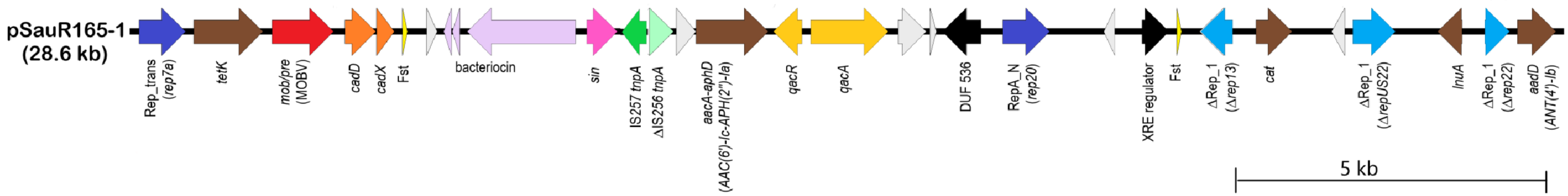

RepA_N + Rep_2 plasmid:

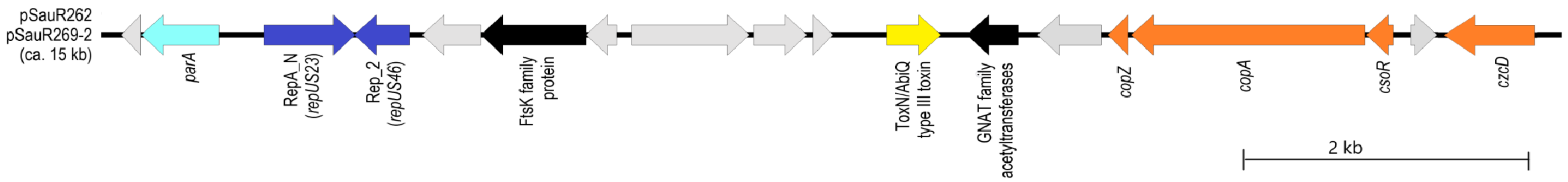

RepA_N + Rep_1 conserved domain plasmids

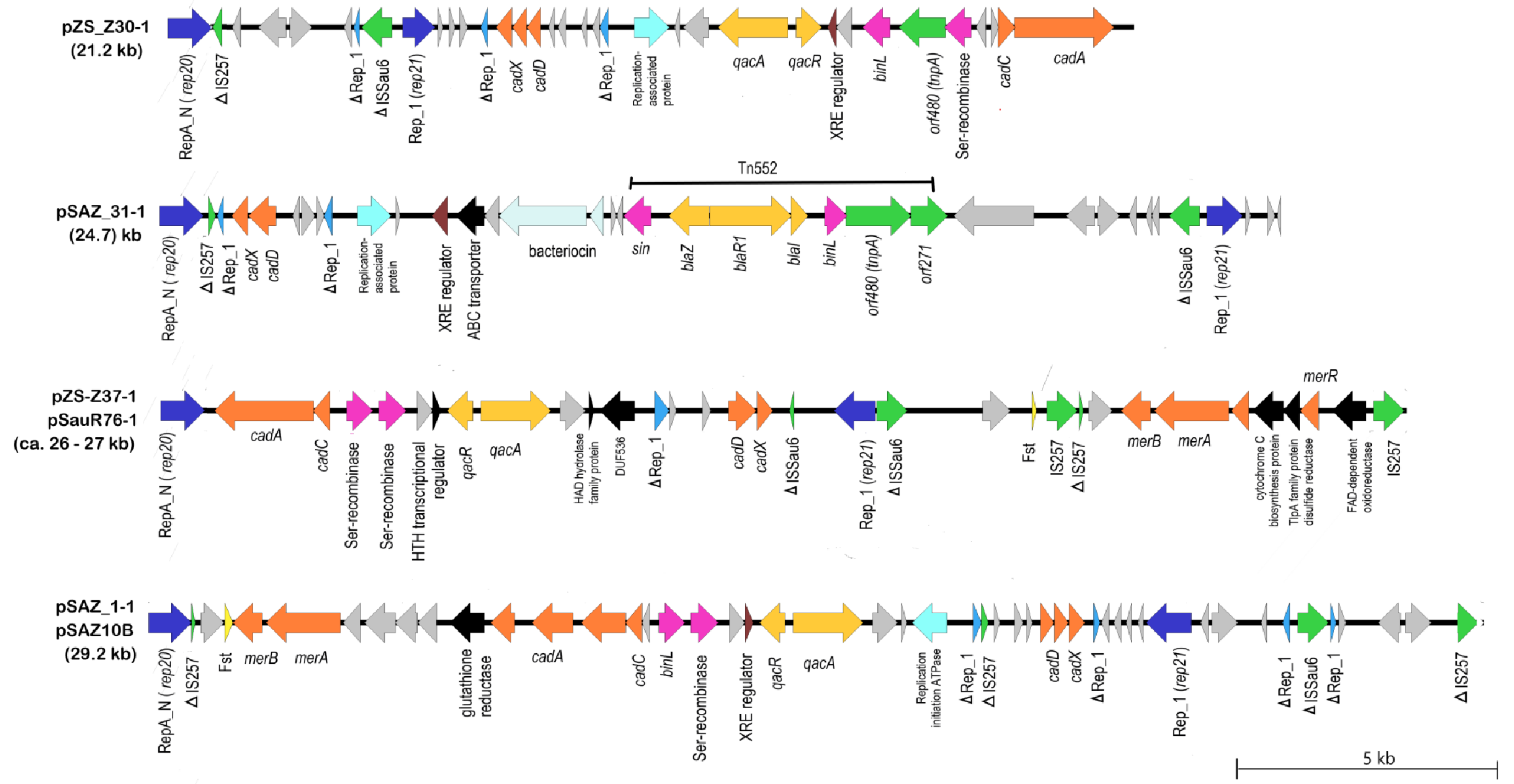

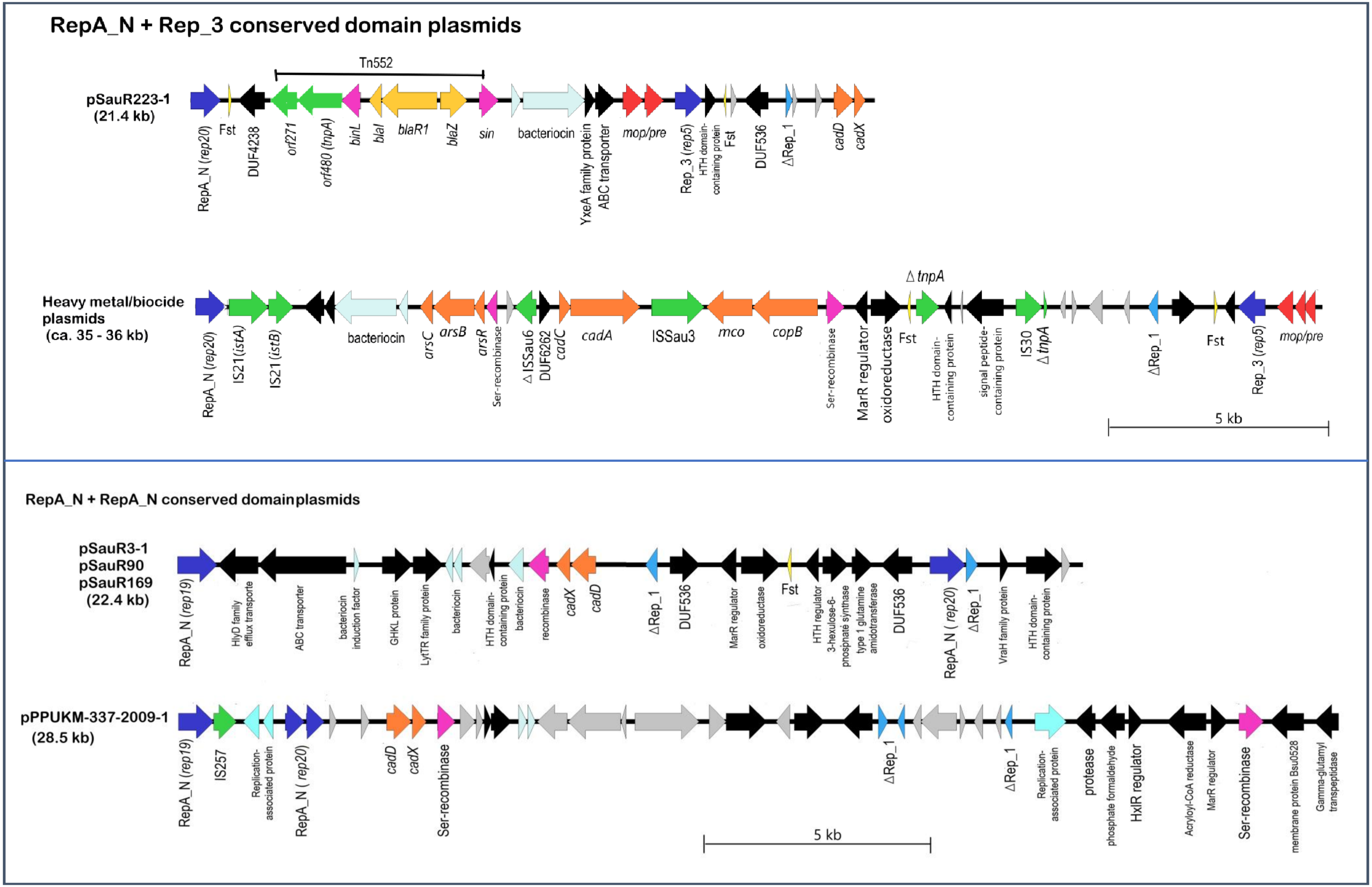
RepA_N + Rep_3 conserved domain plasmids
Tn552
pSauR223-1
(21.4 kb)
RepA_N (rep20)
Fst
DUF4238
orf271
orf480 (tnpA)
binL
blaI
blaR1
blaZ
sin
bacteriocin
YxeA family protein
ABC transporter
mop/pre
Rep_3 (rep5)
HTH domain-containing protein
Fst
DUF536
ΔRep_1
cadD
cadX
Heavy metal/biocide plasmids
(ca. 35 - 36 kb)
RepA_N (rep20)
IS21(istA)
IS21 (istB)
bacteriocin
arsC
arsB
arsR
Ser-recombinase
Δ ISSau6
DUF6262
cadC
cadA
ISSau3
mco
copB
Ser-recombinase
MarR regulator
oxidoreductase
Δ tnpA
Fst
HTH domain-containing protein
signal peptide-containing protein
IS30
Δ tnpA
ΔRep_1
Fst
Rep_3 (rep5)
mop/pre
5 kb
RepA_N + RepA_N conserved domain plasmids
pSauR3-1
pSauR90
pSauR169
(22.4 kb)
RepA_N (rep19)
HlyD family efflux transporte
ABC transporter
bacteriocin induction factor
GHKL protein
LytTR family protein
bacteriocin
HTH domain-containing protein
bacteriocin
recombinase
cadX
cadD
ΔRep_1
DUF536
MarR regulator
oxidoreductase
Fst
HTH regulator
3-hexulose-6-phosphate synthase
type 1 glutamine amidotransferase
DUF536
RepA_N (rep20)
ΔRep_1
VraH family protein
HTH domain-containing protein
pPPUKM-337-2009-1
(28.5 kb)
RepA_N (rep19)
IS257
Replication-associated protein
RepA_N (rep20)
cadD
cadX
Ser-recombinase
ΔRep_1
ΔRep_1
Replication-associated protein
protease
phosphate formaldehyde
HxlR regulator
Acryloyl-CoA reductase
MarR regulator
Ser-recombinase
membrane protein Bsu0528
Gamma-glutamyl transpeptidase
5 kb

Supplementary Figure S1. Linear maps of the plasmid types that were found in this study. **(A)** Maps of single replicon plasmids with the RepL, Rep_1, and Rep_trans domains (i.e., plasmids that replicate using the rolling circle replication (RCR) mechanism); **(B)** single replicon plasmids with the PriCT_1 and Rep_3 conserved domains (plasmids that replicate using the theta replication mode); **(C)** multi-replicon plasmids. Abbreviations: *rep*, replication initiation (replicase) gene; *par*, plasmid partitioning protein-encoding gene; *nes*, relaxase gene; *tra/virD4*, transfer genes; *mobL/mob/pre*, mobilization/recombination genes; *tetK/tetL*, tetracycline resistance genes; *ermC/ermB*, MLS_B resistance genes [*ermC(L)* and *ermB(L)* refer to the leader peptide coding sequence that preceded their respective structural genes and is involved in regulation by attenuation (see main text for details)]; *arsC/arsB/arsR*, arsenic resistance genes; *qacA/qacR*, biocide/antiseptic resistance genes; *cadAC/cadDX*, cadmium resistance genes; *mco*, *copB*, or *copZ*, copper resistance genes; *merA/merB*, mercury resistance genes, *blaZ-blaR1-blaI*, penicillin resistance operon; ANT*(4')-IB* (also designated *aadD*) and *AAC(6')-Ie-APH(2")-Ia* (also designated *aacA-aphD*), aminoglycoside resistance genes; IS, insertion sequence; *sin/bin*: serine recombinase; *mutS*, mismatch DNA repair protein; Fst, toxin of the Fst type I toxin-antitoxin system. Partial or truncated genes or elements are designated with the prefix "Δ". Light grey arrows represent open reading frames (ORFs) for hypothetical proteins; black arrows for ORFs with known domains or functions.
