# Supplementary material for: The Plasmidomic Landscape of Clinical Methicillin-Resistant Staphylococcus aureus Isolates from Malaysia
Source: Antibiotics (Basel). 2023 Apr 9;12(4):733. doi: 10.3390/antibiotics12040733 (PMC10135026; doi:10.3390/antibiotics12040733)
Supplement: Supplementary file 1 [file antibiotics-12-00733-s001.zip › Suppl tables (Table S1 & Table S2).pdf]

Supplementary Table S1. List of plasmids and their genetic characteristics identified in this study along with their respective GenBank accession numbers.

Strain code	Year of isolation / Source of strain	Clonal Complex (CC)	Strain accession no.	Chromosome coverage (average)	Resistance phenotype*	Plasmid name	Size of plasmid in bp	Plasmid accession no.	Plasmid coverage (average)	Replicon type (Conserved domain)	Mobility mechanism(s)	Antimicrobial resistance determinant(s)
SauR3	2016 / blood	CC1	JAIVEG010000000	677.50	FOX, OX, P, CFP, CIP, MXF, E, DA, CN; MDR (5 classes); iMLSB	pSauR3-1	22,425	JAIVEG020000065	750.10	rep19 (RepA_N) + rep20 (RepA_N) +Δrep21 (ΔRep_1)	pWBG749 oriT-OT49 mimic & pWBG749 oriT-OTUNa mimic	none
						pSauR3-2	3,011	JAIVEG010000047.1	3391.8	rep21 (Rep_1)	pWBG749 oriT-OT49 mimic + rep-rlx	none
						pSauR3-3	2,473	JAIVEG020000046.1	3479.2	rep10 (RepL)	none	ermC
SauR7	2016 / pus	NA	JAHGAB000000000	454.6	FOX, OX, P, CFP, CIP, MXF, E, CN, AK, SXT, TE, DO, MH; MDR (6 classes); MS	pSauR7	4,459	JAHGAB010000043.1	3465.2	rep7a (Rep_trans)	Mob/pre (MOBV)	tetK
SauR14	2016 / blood	CC22	JAHKNP000000000	385.7	FOX, OX, P, CFP, CIP, MXF, E, DA; MDR (4 classes); iMLSB	pSauR14-1	35,641	JAHKNP010000031.1	1,415.6	rep20 (RepA_N) + rep5a (Rep_3)	Mob/pre (MOBV)	none
						pSauR14-2	3,050	JAHKNP010000045.1	3408.6	rep21 (Rep_1)	pWBG749 oriT-OT49 mimic+ rep-rlx	none
						pSauR14-3	2,528	JAHKNP010000046.1	3260.7	rep10 (RepL)	None	ermC
SauR23	2016 / pus	CC22	JAIVEH000000000	614.4	FOX, OX, P, CFP, CIP, MXF, E, DA, CN, AK, FD, TE; MDR (7 classes); iMLSB	pSauR23-1	58,422	JAIVEH010000014.1	753.2	repUS20	conjugative	none
						pSauR23-2	3,011	JAIVEH010000068.1	7630.8	rep21 (Rep_1)	pWBG749 oriT-OT49 mimic + rep-rlx	none
						pSauR23-3	2,473	JAIVEH010000073.1	7679.8	rep10 (RepL)	none	ermC
SauR37	2016 / pus	CC1	JAHMGF000000000	922.0	FOX, OX, P, CFP, CIP, MXF, E, CN, AK, SXT; MDR (5 classes); MS	no plasmid detected	-	-	NA	-	-	-
SauR42	2016 / respiratory	CC22	JAHMKI000000000	546.5	FOX, OX, P, CFP, CIP, MXF, E, DA; MDR (4 classes); iMLSB	pSauR42-1	35,337	JAHMKI010000020.1	1063.2	rep20 (RepA_N) + rep5a (Rep_3)	Mob/pre (MOBV)	none
						pSauR42-2	2,600	JAHMKI010000025.1	7749.7	rep10 (RepL)	none	ermC

*Abbreviations used: **β-lactams:** penicillin, P; oxacillin, OX; cefoxitin, FOX; cefoperazone, CFP; **fluoroquinolones**: ciprofloxacin, CIP; moxifloxacin, MXF; **macrolides:** erythromycin, E; **lincosamides:** clindamycin, DA; **aminoglycosides:** gentamicin, CN; amikacin, AK; **folate inhibitors:** co-trimoxazole, SXT; **fucidanes,** fusidic acid, FD; **tetracyclines:** tetracycline, TE; doxycycline, DO; minocycline, MH; **glycylcyclines:** tigecycline, TGC; **phenicols:** chloramphenicol, C; **monocarboxylic acid:** mupirocin, MUP; **ansamycins:** rifampin, RD; **aminocoumarin:** novobiocin, NV; **glycopeptides:** vancomycin, VA; teicoplanin, TEC; **oxazolidinones:** linezolid, LZD; **phosphonic acids**: fosfomycin, FOT; **stretogramins:** quinupristin-dalfopristin, QD; **anti-MRSA cephalosporins:** ceftaroline, CPT. **MLS_B phenotypes**: inducible (I), iMLSB; constitutive (C), cMLSB; macrolide-streptogramin, MS. MDR, multidrug resistance (resistance ≥ 3 classes of antimicrobials).

Strain code	Year of isolation / source of strain	Clonal Complex (CC)	Strain accession no.	Chromosome coverage (average)	Resistance phenotype*	Plasmid name	Size of plasmid in bp	Plasmid accession no.	Plasmid coverage (average)	Replicon type (Conserved domain)	Mobility mechanism(s)	Antimicrobial resistance determinant(s)
SauR46	2016 / pus	CC22	JAHKNQ000000000	424.5	FOX, OX, P, CFP, CIP, MXF, E, DA; MDR (4 classes); cMLSB	pSauR46-1	35,251	JAHKNQ010000030.1	1775.05	rep20 (RepA_N) + rep5a (Rep_3)	Mob/pre (MOBV)	none
						pSauR46-2	3,050	JAHKNQ010000047.1	3452.9	rep21 (Rep_1)	pWBG749 oriT-OT49 mimic + rep-rlx	none
						pSauR46-3	2,422	JAHKNQ010000048.1	3630.6	rep10 (RepL)	none	ermC
SauR47	2016 / pus	CC22	JAHHIP000000000	407.5	FOX, OX, P, CFP, CIP, MXF, E, DA, CN, AK, C; MDR (6 classes); iMLSB	pSauR47-1	36,031	JAHHIP010000022.1	2234.55	rep20 (RepA_N) + rep5a (Rep_3)	Mob/pre (MOBV)	none
						pSauR47-2	4,596	JAHHIP010000027.1	6482.7	rep7a (Rep_trans)	mobCAB/MOBP	cat
						pSauR47-3	3,011	JAHHIP010000031.1	7484.0	rep21 (Rep_1)	pWBG749 oriT-OT49 mimic + rep-rlx	none
						pSauR47-4	2,473	JAHHIP010000033.1	7564.1	rep10 (RepL)	none	ermC
SauR49	2016 / blood	CC22	JAHMGG000000000	613.5	FOX, OX, P, CFP, CIP, MXF, E, DA; MDR (4 classes); iMLSB	pSauR49-1	35,337	JAHMGG010000021.1	1654.0	rep20 (RepA_N) + rep5a (Rep_3)	Mob/pre (MOBV)	none
						pSauR49-2	2,600	JAHMGG010000027.1	7648.6	rep10 (RepL)	none	ermC
SauR53	2016 / blood	CC22	JAHMGH000000000	685.2	FOX, OX, P, CFP, CIP, MXF, E, DA; MDR (4 classes); iMLSB	pSauR53-1	35,337	JAHMGH010000020.1	2.864.8	rep20 (RepA_N) + rep5a (Rep_3)	Mob/pre (MOBV)	none
						pSauR53-2	2,600	JAHMGH010000025.1	7748.6	rep10 (RepL)	none	ermC
SauR57	2016 / pus	CC22	JAIXHC000000000	318.4	FOX, OX, P, CFP, CIP, MXF, E, DA; MDR (4 cl.); iMLSB	pSauR57-1	3,011	JAIXHC010000031.1	6460.1	rep21 (Rep_1)	pWBG749 oriT-OT49 mimic + rep-rlx	none
						pSauR57-2	2,473	JAIXHC010000033.1	5457.5	rep10 (RepL)	none	ermC
SauR58	2016 / respiratory	CC22	JAINVC000000000	312.3	FOX, OX, P, CFP, CIP, MXF, E, DA; MDR (4 classes); cMLSB	pSauR58-1	35,337	JAINVC010000023.1	1280.2	rep20 (RepA_N) + rep5a (Rep_3)	Mob/pre (MOBV)	none
						pSauR58-2	3,138	JAINVC010000028.1	7648.9	rep21 (Rep_1)	pWBG749 oriT-OT49 mimic + rep-rlx	none
						pSauR58-3	2,600	JAINVC010000030.1	7486.9	rep10 (RepL)	none	ermC

*Abbreviations used: **β-lactams:** penicillin, P; oxacillin, OX; cefoxitin, FOX; cefoperazone, CFP; **fluoroquinolones**: ciprofloxacin, CIP; moxifloxacin, MXF; **macrolides:** erythromycin, E; **lincosamides:** clindamycin, DA; **aminoglycosides:** gentamicin, CN; amikacin, AK; **folate inhibitors:** co-trimoxazole, SXT; **fucidanes,** fusidic acid, FD; **tetracyclines:** tetracycline, TE; doxycycline, DO; minocycline, MH; **glycylcylines:** tigecycline, TGC; **phenicols:** chloramphenicol, C; **monocarboxylic acid:** mupirocin, MUP; **ansamycins:** rifampin, RD; **aminocoumarin:** novobiocin, NV; **glycopeptides:** vancomycin, VA; teicoplanin, TEC; **oxazolidinones:** linezolid, LZD; **phosphonic acids**: fosfomycin, FOT; **stretogramins:** quinupristin-dalfopristin, QD; **anti-MRSA cephalosporins:** ceftaroline, CPT. **MLS$_B$ phenotypes**: inducible (I), iMLSB; constitutive (C), cMLSB; macrolide-streptogramin, MS. MDR, multidrug resistance (resistance ≥ 3 classes of antimicrobials).

Strain code	Year of isolation / Source of strain	Clonal Complex (CC)	Strain accession no.	Chromosome coverage (average)	Resistance phenotype*	Plasmid name	Size of plasmid in bp	Plasmid accession no.	Plasmid coverage (average)	Replicon type (Conserved domain)	Mobility mechanism(s)	Antimicrobial resistance determinant(s)
SauR60	2016 / pus	CC22	JAIUDS010000000	366.8	FOX, OX, P, CFP, CIP, MXF, E, DA; MDR (4 classes); iMLSB	pSauR60-1	35,355	JAIUDS010000028.1	1340.9	rep20 (RepA_N) + rep5a (Rep_3)	Mob/pre (MOBV)	none
						pSauR60-2	3,050	JAIUDS010000047.1	6320.3	rep21 (Rep_1)	pWBG749 oriT-OT49 mimic + rep-rlx	none
						pSauR60-3	2,528	JAIUDS010000048.1	4654.7	rep10 (RepL)	none	ermC
SauR63	2016 / pus	CC22	JAIUEA010000000	373.0	FOX, OX, P, CFP, CIP, MXF, E, DA MDR (4 classes); cMLSB	pSauR63-1	35,336	JAIUEA010000032.1	1267.5	rep20 (RepA_N) + rep5a (Rep_3)	Mob/pre (MOBV)	none
						pSauR63-2	3,050	JAIUEA010000049.1	7726.7	rep21 (Rep_1)	pWBG749 oriT-OT49 mimic + rep-rlx	none
						pSauR63-3	2,422	JAIUEA010000050.1	7574.0	rep10 (RepL)	none	ermC
SauR66	2016 / blood	CC22	JAHMGI000000000	686.2	FOX, OX, P, CFP, CIP, MXF, E, DA; MDR (4 classes); iMLSB	pSauR66-1	35,337	JAHMGI010000020.1	2346.6	rep20 (RepA_N) + rep5a (Rep_3)	Mob/pre (MOBV)	none
						pSauR66-2	3,138	JAHMGI010000024.1	7723.5	rep21 (Rep_1)	pWBG749 oriT-OT49 mimic + rep-rlx	none
						pSauR66-3	2,600	JAHMGI010000026.1	7745.7	rep10 (RepL)	none	ermC
SauR68	2016 / blood	CC1	JAHMKB000000000	442.5	FOX, OX, P, CFP, CIP, MXF, E, CN, AK, SXT; MDR (5 classes); MS	No plasmid detected	-	-	NA	-	-	-
SauR69	2016/ pus	CC22	JAIXGZ000000000	332.3	FOX, OX, P, CFP, CIP, MXF, E, DA; MDR (4 classes); iMLSB	pSauR69-1	3,011	JAIXGZ010000031.1	6125.8	rep21 (Rep_1)	pWBG749 oriT-OT49 mimic + rep-rlx	none
						pSauR69-2	2,473	JAIXGZ010000033.1	5260.5	rep10 (RepL)	none	ermC
SauR72	2016/ pus	CC22	JAIXHA000000000	388.0	FOX, OX, P, CFP, CIP, MXF, E, DA; MDR (4 classes); iMLSB	pSauR72-1	3,011	JAIXHA010000026.1	7708.4	rep21 (Rep_1)	pWBG749 oriT-OT49 mimic + rep-rlx	none
						pSauR72-2	2,473	JAIXHA010000029.1	7251.8	rep10 (RepL)	none	ermC
SauR73	2016/ pus	CC22	JAIXHB000000000	381.6	FOX, OX, P, CFP, CIP, MXF, E, DA; MDR (4 classes); iMLSB	pSauR73-1	3,011	JAIXHB010000032.1	7682.1	rep21 (Rep_1)	pWBG749 oriT-OT49 mimic + rep-rlx	none
						pSauR73-2	2,473	JAIXHB010000034.1	7016.0	rep10 (RepL)	none	ermC

*Abbreviations used: **β-lactams:** penicillin, P; oxacillin, OX; cefoxitin, FOX; cefoperazone, CFP; **fluoroquinolones**: ciprofloxacin, CIP; moxifloxacin, MXF; **macrolides:** erythromycin, E; **lincosamides:** clindamycin, DA; **aminoglycosides:** gentamicin, CN; amikacin, AK; **folate inhibitors:** co-trimoxazole, SXT; **fucidanes,** fusidic acid, FD; **tetracyclines:** tetracycline, TE; doxycycline, DO; minocycline, MH; **glycylcyclines:** tigecycline, TGC; **phenicols:** chloramphenicol, C; **monocarboxylic acid:** mupirocin, MUP; **ansamycins:** rifampin, RD; **aminocoumarin:** novobiocin, NV; **glycopeptides:** vancomycin, VA; teicoplanin, TEC; **oxazolidinones:** linezolid, LZD; **phosphonic acids**: fosfomycin, FOT; **stretogramins:** quinupristin-dalfopristin, QD; **anti-MRSA cephalosporins:** ceftaroline, CPT. **MLS_B phenotypes**: inducible (I), iMLSB; constitutive (C), cMLSB; macrolide-streptogramin, MS. MDR, multidrug resistance (resistance ≥ 3 classes of antimicrobials).

Strain code	Year of isolation / Source of strain	Clonal Complex (CC)	Strain accession no.	Chromosome coverage (average)	Resistance phenotype*	Plasmid name	Size of plasmid in bp	Plasmid accession no.	Plasmid coverage (average)	Replicon type (Conserved domain)	Mobility mechanism(s)	Antimicrobial resistance determinant(s)
SauR74	2016/ pus	CC22	JAHMKC000000000	490.2	FOX, OX, P, CFP, CIP, MXF, E, DA, TGC; MDR (5 classes); iMLSB	pSauR74-1	3,011	JAHMKC010000030.1	7647.4	rep21 (Rep_1)	pWBG749 oriT-OT49 mimic + rep-rlx	none
						pSauR74-2	2,473	JAHMKC010000033.1	7508.6	rep10 (RepL)	none	ermC
SauR76	2016/ blood	CC8	JAHMKD000000000	357.3	FOX, OX, P, CFP, CIP, MXF, E, DA, CN, AK, SXT, FD, TE, DO, MH, RD; MDR (9 classes); cMLSB	pSauR76-1	27,579	JAHMKD010000027.1	1281..0	rep20 (RepA_N) + rep21 (Rep_1)	pWBG749 oriT-OTUNa & pWBG749 oriT-OT49 mimic + rep-rlx	none
						pSauR76-2	4,459	JAHMKD010000042.1	3378.7	rep7a (Rep_trans)	Mob/pre (MOBV)	tetK
SauR77	2016/ blood	CC22	JAHMGJ000000000	660.8	FOX, OX, P, CFP, CIP, MXF, E, DA; MDR (4 classes); iMLSB	pSauR77-1	35,618	JAHMGJ010000029.1	2068.3	rep20 (RepA_N) + rep5a (Rep_3)	Mob/pre (MOBV)	None
						pSauR77-2	2,550	JAHMGJ010000039.1	7437.6	rep10 (RepL)	none	ermC
SauR78	2016/ pus	CC22	JAIUDT010000000	376.6	FOX, OX, P, CFP, CIP, MXF, E, DA; MDR (4 classes); iMLSB	pSauR78-1	35,336	JAIUDT010000029.1	1201.6	rep20 (RepA_N) + rep5a (Rep_3)	Mob/pre (MOBV)	none
						pSauR78-2	2,528	JAIUDT010000049.1	5436.3	rep10 (RepL)	none	ermC
SauR81	2017/ blood	CC22	JAHMGK000000000	686.8	FOX, OX, P, CFP, CIP, MXF, E, DA; MDR (4 classes); iMLSB	pSauR81-1	36,054	JAHMGK010000024.1	2603.9	rep20 (RepA_N) + rep5a (Rep_3)	Mob/pre (MOBV)	none
						pSauR81-2	3,088	JAHMGK010000033.1	7672.6	rep21 (Rep_1)	pWBG749 oriT-OT49 mimic + rep-rlx	none
						pSauR81-3	2,550	JAHMGK010000035.1	7698.4	rep10 (RepL)	none	ermC
SauR82	2017/ blood	CC22	JAHMGL000000000	723.3	FOX, OX, P, CFP, CIP, MXF, E, DA; MDR (4 classes); iMLSB	pSauR82-1	35,415	JAHMGL010000026.1	3049.3	rep20 (RepA_N) + rep5a (Rep_3)	Mob/pre (MOBV)	none
						pSauR82-2	3,088	JAHMGL010000034.1	7772.7	rep21 (Rep_1)	pWBG749 oriT-OT49 mimic + rep-rlx	none
						pSauR82-3	2,550	JAHMGL010000036.1	7751.0	rep10 (RepL)	none	ermC
SauR83	2017/ blood	CC22	JAHMGM000000000	576.1	FOX, OX, P, CFP, CIP, MXF, E, DA; MDR (4 classes); iMLSB	pSauR83-1	3,011	JAHMGM010000026.1	7667.3	rep21 (Rep_1)	pWBG749 oriT-OT49 mimic + rep-rlx	none
						pSauR83-2	2,473	JAHMGM010000028.1	7615.8	rep10 (RepL)	none	ermC

*Abbreviations used: **β-lactams:** penicillin, P; oxacillin, OX; cefoxitin, FOX; cefoperazone, CFP; **fluoroquinolones**: ciprofloxacin, CIP; moxifloxacin, MXF; **macrolides:** erythromycin, E; **lincosamides:** clindamycin, DA; **aminoglycosides:** gentamicin, CN; amikacin, AK; **folate inhibitors:** co-trimoxazole, SXT; **fucidanes,** fusidic acid, FD; **tetracyclines:** tetracycline, TE; doxycycline, DO; minocycline, MH; **glycylcylines:** tigecycline, TGC; **phenicols:** chloramphenicol, C; **monocarboxylic acid:** mupirocin, MUP; **ansamycins:** rifampin, RD; **aminocoumarin:** novobiocin, NV; **glycopeptides:** vancomycin, VA; teicoplanin, TEC; **oxazolidinones:** linezolid, LZD; **phosphonic acids**: fosfomycin, FOT; **stretogramins:** quinupristin-dalfopristin, QD; **anti-MRSA cephalosporins:** ceftaroline, CPT. **MLS_B phenotypes**: inducible (I), iMLSB; constitutive (C), cMLSB; macrolide-streptogramin, MS. MDR, multidrug resistance (resistance ≥ 3 classes of antimicrobials).

Strain code	Year of isolation / Source of strain	Clonal Complex (CC)	Strain accession no.	Chromosome coverage (average)	Resistance phenotype*	Plasmid name	Size of plasmid in bp	Plasmid accession no.	Plasmid coverage (average)	Replicon type (Conserved domain)	Mobility mechanism(s)	Antimicrobial resistance determinant(s)
SauR84	2017/ blood	CC22	JAHMGN000000000	534.6	FOX, OX, P, CFP, CIP, MXF, E, DA, C; MDR (5 classes); iMLSB	pSauR84-1	3,733	JAHMGN010000028.1	7666.6	rep7a (Rep_trans)	Mob/pre (MOBV)	cat
						pSauR84-2	3,011	JAHMGN010000032.1	7671.6	rep21 (Rep_1)	pWBG749 oriT-OT49 mimic + rep-rlx	none
						pSauR84-3	2,473	JAHMGN010000034.1	7627.6	rep10 (RepL)	none	ermC
SauR85	2017/ blood	CC22	JAHMKE000000000	471.7	FOX, OX, P, CFP, CIP, MXF, E, DA, C; MDR (5 classes); iMLSB	pSauR85-1	3,733	JAHMKE010000028.1	7716.7	rep7a (Rep_trans)	Mob/pre (MOBV)	cat
						pSauR85-2	3,011	JAHMKE010000032.1	7654.4	rep21 (Rep_1)	pWBG749 oriT-OT49 mimic + rep-rlx	none
						pSauR85-3	2,473	JAHMKE010000034.1	7711.9	rep10 (RepL)	none	ermC
SauR86	2017/ blood	CC22	JAHMGT000000000	585.7	FOX, OX, P, CFP, CIP, MXF, E, DA; MDR (4 classes); iMLSB	pSauR86-1	35,640	JAHMGT010000022.1	2616.5	rep20 (RepA_N) + rep5a (Rep_3)	Mob/pre (MOBV)	none
						pSauR86-2	3,138	JAHMGT010000029.1	7665.6	rep21 (Rep_1)	pWBG749 oriT-OT49 mimic + rep-rlx	none
						pSauR86-3	2,600	JAHMGT010000031.1	7724.8	rep10 (RepL)	none	ermC
SauR88	2017/ pus	CC22	JAHMKF000000000	402.8	FOX, OX, P, CFP, CIP, MXF, E, DA, TGC; MDR (5 classes); iMLSB	pSauR88-1	3,011	JAHMKF010000027.1	7655.3	rep21 (Rep_1)	pWBG749 oriT-OT49 mimic + rep-rlx	none
						pSauR88-2	2,473	JAHMKF010000029.1	7589.2	rep10 (RepL)	none	ermC
SauR90	2017/ blood	CC22	JAIVEI000000000	660.3	FOX, OX, P, E, CN; MDR (3 classes); MS	pSauR90	22,435	JAIVEI020000038	728.5	rep19 (RepA_N) rep20 (RepA_N) +Δrep21 (Rep_1)	pWBG749 oriT-OT49 mimic & pWBG749 oriT-OTUNa mimic	none
SauR91	2017/ respiratory	CC22	JAHMGR000000000	529.7	FOX, OX, P, CFP, CIP, MXF, E, DA; MDR (4 classes); iMLSB	pSauR91-1	35,640	JAHMGR010000023.1	2140.9	rep20 (RepA_N) + rep5a (Rep_3)	Mob/pre (MOBV)	none
						pSauR91-2	3,138	JAHMGR010000027.1	7676.2	rep21 (Rep_1)	pWBG749 oriT-OT49 mimic + rep-rlx	none
SauR92	2017/ respiratory	CC22	JAHMGS000000000	570.4	FOX, OX, P, CFP, CIP, MXF, E, DA; MDR (4 classes); iMLSB	pSauR92-1	35,337	JAHMGS010000022.1	1742.0	rep20 (RepA_N) + rep5a (Rep_3)	Mob/pre (MOBV)	none
						pSauR92-2	3,138	JAHMGS010000028.1	7563.9	rep21 (Rep_1)	pWBG749 oriT-OT49 mimic + rep-rlx	none
						pSauR92-3	2,600	JAHMGS010000031.1	7512.0	rep10 (RepL)	none	ermC

*Abbreviations used: **β-lactams:** penicillin, P; oxacillin, OX; cefoxitin, FOX; cefoperazone, CFP; **fluoroquinolones**: ciprofloxacin, CIP; moxifloxacin, MXF; **macrolides:** erythromycin, E; **lincosamides:** clindamycin, DA; **aminoglycosides:** gentamicin, CN; amikacin, AK; **folate inhibitors:** co-trimoxazole, SXT; **fucidanes,** fusidic acid, FD; **tetracyclines:** tetracycline, TE; doxycycline, DO; minocycline, MH; **glycylcylines:** tigecycline, TGC; **phenicols:** chloramphenicol, C; **monocarboxylic acid:** mupirocin, MUP; **ansamycins:** rifampin, RD; **aminocoumarin:** novobiocin, NV; **glycopeptides:** vancomycin, VA; teicoplanin, TEC; **oxazolidinones:** linezolid, LZD; **phosphonic acids**: fosfomycin, FOT; **stretogramins:** quinupristin-dalfopristin, QD; **anti-MRSA cephalosporins:** ceftaroline, CPT. **MLS_B phenotypes**: inducible (I), iMLSB; constitutive (C), cMLSB; macrolide-streptogramin, MS. MDR, multidrug resistance (resistance ≥ 3 classes of antimicrobials).

Strain code	Year of isolation / Source of strain	Clonal Complex (CC)	Strain accession no.	Chromosome coverage (average)	Resistance phenotype*	Plasmid name	Size of plasmid in bp	Plasmid accession no.	Plasmid coverage (average)	Replicon type (Conserved domain)	Mobility mechanism(s)	Antimicrobial resistance determinant(s)
SauR93	2017/ blood	CC22	JAHMGO000000000	740.8	FOX, OX, P, CFP, CIP, MXF, E, DA; MDR (4 classes); cMLSB	pSauR93-1	35,237	JAHMGO010000024.1	2726.3	rep20 (RepA_N) + rep5a (Rep_3)	Mob/pre (MOBV)	none
						pSauR93-2	3,088	JAHMGO010000032.1	7685.4	rep21 (Rep_1)	pWBG749 oriT-OT49 mimic + rep-rlx	none
						pSauR93-3	2,444	JAHMGO010000035.1	7601.2	rep10 (RepL)	none	ermC
SauR103	2017/ pus	CC22	JAHMKG000000000	501.4	FOX, OX, P, CFP, CIP, MXF, E, DA, TGC; MDR (5 classes); iMLSB	pSauR103-1	35,337	JAHMKG010000020.1	1737.6	rep20 (RepA_N) + rep5a (Rep_3)	Mob/pre (MOBV)	none
						pSauR103-2	2,600	JAHMKG010000025.1	7613.9	rep10 (RepL)	none	ermC
SauR105	2017/ blood	CC22	JAHMGP000000000	655.4	FOX, OX, P, CFP, CIP, MXF, E, DA; MDR (4 classes); cMLSB	pSauR105-1	35,656	JAHMGP010000027.1	2435.7	rep20 (RepA_N) + rep5a (Rep_3)	Mob/pre (MOBV)	none
						pSauR105-2	3,088	JAHMGP010000042.1	7694.5	rep21 (Rep_1)	pWBG749 oriT-OT49 mimic + rep-rlx	none
						pSauR105-3	2,444	JAHMGP010000044.1	7599.2	rep10 (RepL)	none	ermC
SauR106	2017/ blood	CC22	JAHMGQ000000000	540.8	FOX, OX, P, CFP, CIP, MXF, E, DA; MDR (4 classes); iMLSB	pSauR106-1	35,316	JAHMGQ010000025.1	2018.3	rep20 (RepA_N) + rep5a (Rep_3)	Mob/pre (MOBV)	none
						pSauR106-2	3,088	JAHMGQ010000037.1	7742.8	rep21 (Rep_1)	pWBG749 oriT-OT49 mimic + rep-rlx	none
						pSauR106-3	2,550	JAHMGQ010000038.1	7751.3	rep10 (RepL)	none	ermC
SauR118	2017/ pus	CC22	JAHMKH000000000	483.0	FOX, OX, P, CFP, CIP, MXF, E, DA, TGC; MDR (5 classes); iMLSB	pSauR118-1	35,337	JAHMKH010000021.1	1540.3	rep20 (RepA_N) + rep5a (Rep_3)	Mob/pre (MOBV)	none
						pSauR118-2	3,138	JAHMKH010000025.1	7665.4	rep21 (Rep_1)	pWBG749 oriT-OT49 mimic + rep-rlx	none
						pSauR118-3	2,600	JAHMKH010000027.1	7563.6	rep10 (RepL)	none	ermC
SauR127	2017/ pus	CC1	JAIVEJ000000000	406.1	FOX, OX, P, FD, CN, AK, TE, DO, TGC; MDR (5 classes)	pSauR127	25,167	JAIVEJ010000022.1	2207.0	rep5a (Rep_3) + Δrep22 (Rep_1) Δrep13 (Rep_1)	Mob/pre (MOBV)	bla operon + tetL

*Abbreviations used: **β-lactams:** penicillin, P; oxacillin, OX; cefoxitin, FOX; cefoperazone, CFP; **fluoroquinolones**: ciprofloxacin, CIP; moxifloxacin, MXF; **macrolides:** erythromycin, E; **lincosamides:** clindamycin, DA; **aminoglycosides:** gentamicin, CN; amikacin, AK; **folate inhibitors:** co-trimoxazole, SXT; **fucidanes,** fusidic acid, FD; **tetracyclines:** tetracycline, TE; doxycycline, DO; minocycline, MH; **glycylcylines:** tigecycline, TGC; **phenicols:** chloramphenicol, C; **monocarboxylic acid:** mupirocin, MUP; **ansamycins:** rifampin, RD; **aminocoumarin:** novobiocin, NV; **glycopeptides:** vancomycin, VA; teicoplanin, TEC; **oxazolidinones:** linezolid, LZD; **phosphonic acids**: fosfomycin, FOT; **stretogramins:** quinupristin-dalfopristin, QD; **anti-MRSA cephalosporins:** ceftaroline, CPT. **MLS_B phenotypes**: inducible (I), iMLSB; constitutive (C), cMLSB; macrolide-streptogramin, MS. MDR, multidrug resistance (resistance ≥ 3 classes of antimicrobials).

Supplementary Table S1

Strain code	Year of isolation / Source of strain	Clonal Complex (CC)	Strain accession no.	Chromosome coverage (average)	Resistance phenotype*	Plasmid name	Size of plasmid in bp	Plasmid accession no.	Plasmid coverage (average)	Replicon type (Conserved domain)	Mobility mechanism(s)	Antimicrobial resistance determinant(s)
SauR133	2017/ blood	CC22	JAHMGU000000000	382.4	FOX, OX, P, CFP, CIP, MXF, E, DA; MDR (4 classes); iMLSB	pSauR133-1	34,958	JAHMGU010000031.1	1775.2	rep20 (RepA_N) + rep5a (Rep_3)	Mob/pre (MOBV)	none
						pSauR133-2	3,050	JAHMGU010000048.1	1428.5	rep21 (Rep_1)	pWBG749 oriT-OT49 mimic + rep-rlx	none
						pSauR133-3	2,518	JAHMGU010000049.1	1101.6	rep10 (RepL)	none	ermC
SauR142	2017/ respiratory	CC22	JAHMGV000000000	382.4	FOX, OX, P, CFP, CIP, MXF, E, DA; MDR (4 classes); iMLSB	pSauR142-1	35,604	JAHMGV010000031.1	1775.2	rep20 (RepA_N) + rep5a (Rep_3)	Mob/pre (MOBV)	none
						pSauR142-2	3,050	JAHMGV010000048.1	1428.5	rep21 (Rep_1)	pWBG749 oriT-OT49 mimic + rep-rlx	none
						pSauR142-3	2,518	JAHMGV010000049.1	1101.6	rep10 (RepL)	none	ermC
SauR143	2017/ blood	CC22	JAHMGW000000000	393.6	FOX, OX, P, CFP, CIP, MXF, E, DA; MDR (4 classes); iMLSB	pSauR143-1	35,674	JAHMGW010000027.1	1817.9	rep20 (RepA_N) + rep5a (Rep_3)	Mob/pre (MOBV)	none
						pSauR143-2	3,050	JAHMGW010000046.1	2002.6	rep21 (Rep_1)	pWBG749 oriT-OT49 mimic + rep-rlx	none
						pSauR143-3	2,528	JAHMGW010000047.1	1814.6	rep10 (RepL)	none	ermC
SauR144	2017/ blood	CC22	JAHMGX000000000	393.6	FOX, OX, P, CFP, CIP, MXF, E, DA; MDR (4 classes); iMLSB	pSauR144-1	35,332	JAHMGX010000027.1	1817.9	rep20 (RepA_N) + rep5a (Rep_3)	Mob/pre (MOBV)	none
						pSauR144-2	3,050	JAHMGX010000046.1	2002.6	rep21 (Rep_1)	pWBG749 oriT-OT49 mimic + rep-rlx	none
						pSauR144-3	2,528	JAHMGX010000047.1	1814.6	rep10 (RepL)	none	ermC
SauR156	2017/ pus	CC1	JAIUJG000000000	365.7	FOX, OX, P, DA, E, SXT, CN, AK, TE, DO, CIP, MXF, C; MDR (8 classes); cMLSB	pSauR156-1	11341	JAIUJG010000020.1	1507.3	repUS18 (PriCT-1)	Mob/pre (MOBV) (2 copies)	ermB, tetL ant(4')-Ib
						pSauR156-2	3,010	JAIUJG010000029.1	2056.9	rep13 (Rep_1)	rep-rlx	none
						pSauR156-3	2,886	JAIUJG010000031	1368.4	rep21 (Rep_1)	rep-rlx	none
SauR157	2017/ pus	CC22	JAHMGY000000000	377.2	FOX, OX, P, CFP, CIP, MXF, E, DA, FD; MDR (5 classes); iMLSB	pSauR157-1	3,011	JAHMGY010000028.1	1919.6	rep21 (Rep_1)	pWBG749 oriT-OT49 mimic + rep-rlx	none
						pSauR157-2	2,473	JAHMGY010000030.1	1332.0	rep10 (RepL)	none	ermC

*Abbreviations used: **β-lactams:** penicillin, P; oxacillin, OX; cefoxitin, FOX; cefoperazone, CFP; **fluoroquinolones**: ciprofloxacin, CIP; moxifloxacin, MXF; **macrolides:** erythromycin, E; **lincosamides:** clindamycin, DA; **aminoglycosides:** gentamicin, CN; amikacin, AK; **folate inhibitors:** co-trimoxazole, SXT; **fucidanes,** fusidic acid, FD; **tetracyclines:** tetracycline, TE; doxycycline, DO; minocycline, MH; **glycylcylines:** tigecycline, TGC; **phenicols:** chloramphenicol, C; **monocarboxylic acid:** mupirocin, MUP; **ansamycins:** rifampin, RD; **aminocoumarin:** novobiocin, NV; **glycopeptides:** vancomycin, VA; teicoplanin, TEC; **oxazolidinones:** linezolid, LZD; **phosphonic acids**: fosfomycin, FOT; **stretogramins:** quinupristin-dalfopristin, QD; **anti-MRSA cephalosporins:** ceftaroline, CPT. **MLS_B phenotypes**: inducible (I), iMLSB; constitutive (C), cMLSB; macrolide-streptogramin, MS. MDR, multidrug resistance (resistance ≥ 3 classes of antimicrobials).

Strain code	Year of isolation / Source of strain	Clonal Complex (CC)	Strain accession no.	Chromosome coverage (average)	Resistance phenotype*	Plasmid name	Size of plasmid in bp	Plasmid accession no.	Plasmid coverage (average)	Replicon type (Conserved domain)	Mobility mechanism(s)	Antimicrobial resistance determinant(s)
SauR165	2017/ blood	CC22	JAHMGZ000000000	349.5	FOX, OX, P, CFP, CIP, MXF, E, DA, AK, CN, TE, C; MDR (7 classes); iMLSB	pSauR165-1	28,649	JAHMGZ010000022.1	1021.6	rep7a(Rep_trans) + rep20 (RepA_N) +ΔrepUS22 (ΔRep_1)+ Δrep22(ΔRep_1)+ Δrep13(ΔRep_1)	Mob/pre (MOBV)	tetK, lnuA, cat aac(6')-Ie-aph(2")-Ia ant(4')-Ib
						pSauR165-2	3,011	JAHMGZ010000027.1	3360.1	rep21 (Rep_1)	pWBG749 oriT-OT49 mimic + rep-rlx	none
						pSauR165-3	2,473	JAHMGZ010000029.1	3274.1	rep10 (RepL)	none	ermC
SauR168	2017/ pus	CC1	JAHMHA000000000	361.0	FOX, OX, P, CFP, CIP, MXF, E, DA, CN, FD; MDR (6 classes); iMLSB	pSauR168-1	20,652	JAHMHA010000015.1	867.8	rep5a (rep_3)	Mob/pre (MOBV)	bla operon
						pSauR168-2	2,473	JAHMHA010000020.1	3154.9	rep10 (RepL)	none	ermC
SauR169	2017/ pus	CC1	JAIVEK000000000	376.1	FOX, P, E, CN, FD, NV; MDR (5 classes); MS	pSauR169	22,490	JAIVEK020000054	970.3	rep19 (RepA_N) +rep20 (RepA_N) Δrep21 (rep_1)	pWBG749 oriT-OT49 mimic & pWBG749 oriT-OTUNa mimic	none
SauR171	2017/ pus	CC22	JAHMHB000000000	364.2	FOX, OX, P, CIP, MXF, E, DA, FD. MDR (5 classes); iMLSB	pSauR171-1	3,011	JAHMHB010000026.1	1915.5	rep21 (Rep_1)	pWBG749 oriT-OT49 mimic + rep-rlx	none
						pSauR171-2	2,473	JAHMHB010000028.1	1362.5	rep10 (RepL)	none	ermC
SauR176	2017/ respiratory	CC22	JAHMHC000000000	377.4	FOX, OX, P, CIP, E, DA. MDR (4 classes); iMLSB	pSauR176-1	3,011	JAHMHC010000031.1	1003.7	rep21 (Rep_1)	pWBG749 oriT-OT49 mimic + rep-rlx	none
						pSauR176-2	2,463	JAHMHC010000033.1	1597.7	rep10 (RepL)	none	ermC
						pSauR176-3	2,397	JAHMHC010000034.1	1244.6	repUS21 (Rep_1)	rep-rlx	none
SauR181	2018/ pus	CC22	JAHMHD000000000	369.0	FOX, OX, P, CIP, MXF, E, DA , FD; MDR (5 classes); iMLSB	pSauR181-1	35,356	JAHMHD010000029.1	1754.0	rep20 (RepA_N) + rep5a (Rep_3)	Mob/pre (MOBV)	none
						pSauR181-2	3,050	JAHMHD010000044.1	3834.8	rep21 (Rep_1)	pWBG749 oriT-OT49 mimic + rep-rlx	none
						pSauR181-3	2,528	JAHMHD010000045.1	3645.7	rep10 (RepL)	none	ermC

*Abbreviations used: **β-lactams:** penicillin, P; oxacillin, OX; cefoxitin, FOX; cefoperazone, CFP; **fluoroquinolones**: ciprofloxacin, CIP; moxifloxacin, MXF; **macrolides:** erythromycin, E; **lincosamides:** clindamycin, DA; **aminoglycosides:** gentamicin, CN; amikacin, AK; **folate inhibitors:** co-trimoxazole, SXT; **fucidanes,** fusidic acid, FD; **tetracyclines:** tetracycline, TE; doxycycline, DO; minocycline, MH; **glycylcyclines:** tigecycline, TGC; **phenicols:** chloramphenicol, C; **monocarboxylic acid:** mupirocin, MUP; **ansamycins:** rifampin, RD; **aminocoumarin:** novobiocin, NV; **glycopeptides:** vancomycin, VA; teicoplanin, TEC; **oxazolidinones:** linezolid, LZD; **phosphonic acids**: fosfomycin, FOT; **stretogramins:** quinupristin-dalfopristin, QD; **anti-MRSA cephalosporins:** ceftaroline, CPT. **MLS_B phenotypes**: inducible (I), iMLSB; constitutive (C), cMLSB; macrolide-streptogramin, MS. MDR, multidrug resistance (resistance ≥ 3 classes of antimicrobials).

Strain code	Year of isolation / Source of strain	Clonal Complex (CC)	Strain accession no.	Chromosome coverage (average)	Resistance phenotype*	Plasmid name	Size of plasmid in bp	Plasmid accession no.	Plasmid coverage (average)	Replicon type (Conserved domain)	Mobility mechanism(s)	Antimicrobial resistance determinant(s)
SauR192	2019/ blood	CC22	JAIUDU010000000	330.9	FOX, OX, P, CFP, CIP, MXF, E, DA; MDR (4 classes); iMLSB	pSauR192-1	35,070	JAIUDU010000030.1	1456.3	rep20 (RepA_N) + rep5a (Rep_3)	Mob/pre (MOBV)	none
						pSauR192-2	3,050	JAIUDU010000048.1	5393.1	rep21 (Rep_1)	pWBG749 oriT-OT49 mimic + rep-rlx	none
						pSauR192-3	2,528	JAIUDU010000049.1	4520.6	rep10 (RepL)	none	ermC
SauR193	2019/ blood	NA	JAIWLV000000000	340.4	FOX, OX, P, CIP, MXF, CN; MDR (3 classes)	pSauR193	20,727	JAIWLV010000015.1	1404.5	rep5a (rep_3)	Mob/pre (MOBV)	bla operon
SauR200	2019/ pus	CC22	JAKRCL000000000	413.3	FOX, OX, P, CIP, MXF, E, DA; MDR (4 classes); iMLSB	pSauR200-1	35,336	JAKRCL010000072	1851.0	rep20 (RepA_N) + rep5a (Rep_3)	Mob/pre (MOBV)	none
						pSauR200-2	2,528	JAKRCL010000045	3662.6	rep10 (RepL)	none	ermC
SauR203	2019/ pus	CC22	JAKRCM000000000	417.9	FOX, OX, P, CIP, MXF, E, DA; MDR (4 classes); iMLSB	pSauR203-1	35,336	JAKRCM010000064	1806.2	rep20 (RepA_N) + rep5a (Rep_3)	Mob/pre (MOBV)	none
						pSauR203-2	2,528	JAKRCM010000045	3338.1	rep10 (RepL)	none	ermC
SauR204	2019/ tissue	CC22	JAIUDV010000000	361.8	FOX, OX, P, CFP, CIP, MXF, E, DA; MDR (4 classes); iMLSB	pSauR204-1	35,286	JAIUDV010000031.1	1993.3	rep20 (RepA_N) + rep5a (Rep_3)	Mob/pre (MOBV)	none
						pSauR204-2	2,528	JAIUDV010000045.1	2391.9	rep10 (RepL)	none	ermC
SauR206	2019/ pus	CC22	JAIUDW010000000	377.8	FOX, OX, P, CFP, CIP, MXF, E, DA; MDR (4 classes); iMLSB	pSauR206-1	35,081	JAIUDW010000030.1	1891.3	rep20 (RepA_N) + rep5a (Rep_3)	Mob/pre (MOBV)	none
						pSauR206-2	2,528	JAIUDW010000044.1	2268.2	rep10 (RepL)	none	ermC
SauR207	2019/ blood	CC1	JAHMHE000000000	351.5	FOX, P, CIP, MXF, E, DA, CN, SXT, FD; MDR (7 classes); iMLSB	pSauR207-1	20,652	JAHMHE010000012.1	1223.2	rep5a (Rep_3)	Mob/pre (MOBV)	bla operon
						pSauR207-2	2,473	JAHMHE010000017.1	1807.6	rep10 (RepL)	none	ermC
SauR209	2019/ pus	CC1	JAHOPA000000000	345.0	FOX, OX, P, CN, FD, TE, DO; MDR (4 classes)	pSauR209	25,167	JAHOPA010000017	1718.7	rep5a (Rep_3) + Δrep13 (ΔRep_1)	Mob/pre (MOBV)	bla operon+ tetL
SauR210	2019/ blood	CC22	JAIXHD000000000	447.6	FOX, OX, P, CFP, CIP, MXF, E, DA; MDR (4 classes); iMLSB	pSauR210-1	3,011	JAIXHD010000034.1	1885.0	rep21 (Rep_1)	pWBG749 oriT-OT49 mimic + rep-rlx	none
						pSauR210-2	2,473	JAIXHD010000036.1	1374.7	rep10 (RepL)	none	ermC
SauR211	2019/ pus	CC22	JAIXHE000000000	347.3	FOX, OX, P, CFP, CIP, MXF, RD; MDR (3 classes)	pSauR211	3011	JAIXHE010000033.1	2214.2	rep21 (Rep_1)	pWBG749 oriT-OT49 mimic + rep-rlx	none

*Abbreviations used: **β-lactams:** penicillin, P; oxacillin, OX; cefoxitin, FOX; cefoperazone, CFP; **fluoroquinolones**: ciprofloxacin, CIP; moxifloxacin, MXF; **macrolides:** erythromycin, E; **lincosamides:** clindamycin, DA; **aminoglycosides:** gentamicin, CN; amikacin, AK; **folate inhibitors:** co-trimoxazole, SXT; **fucidanes,** fusidic acid, FD; **tetracyclines:** tetracycline, TE; doxycycline, DO; minocycline, MH; **glycylcylines:** tigecycline, TGC; **phenicols:** chloramphenicol, C; **monocarboxylic acid:** mupirocin, MUP; **ansamycins:** rifampin, RD; **aminocoumarin:** novobiocin, NV; **glycopeptides:** vancomycin, VA; teicoplanin, TEC; **oxazolidinones:** linezolid, LZD; **phosphonic acids**: fosfomycin, FOT; **stretogramins:** quinupristin-dalfopristin, QD; **anti-MRSA cephalosporins:** ceftaroline, CPT. **MLS_B phenotypes**: inducible (I), iMLSB; constitutive (C), cMLSB; macrolide-streptogramin, MS. MDR, multidrug resistance (resistance ≥ 3 classes of antimicrobials).

Strain code	Year of isolation / Source of strain	Clonal Complex (CC)	Strain accession no.	Chromosome coverage (average)	Resistance phenotype*	Plasmid name	Size of plasmid in bp	Plasmid accession no.	Plasmid coverage (average)	Replicon type (Conserved domain)	Mobility mechanism(s)	Antimicrobial resistance determinant(s)
SauR221	2019/ pus	CC1	JAHMHF000000000	312.7	FOX, P, CIP, CN, MXF, E, DA, FD; MDR (6 classes); iMLSB	pSauR221-1	20,652	JAHMHF010000012.1	1128.7	rep5a (Rep_3)	Mob/pre (MOBV)	bla operon
						pSauR221-2	2,473	JAHMHF010000016.1	3857.3	rep10 (RepL)	none	ermC
SauR222	2019/ blood	CC22	JAIUEB010000000	433.7	FOX, OX, P, CFP, CIP, E, DA; MDR (4 classes) cMLSb	pSauR222-1	35,286	JAIUEB010000029.1	1614.2	rep20 (RepA_N) + rep5a (Rep_3)	Mob/pre (MOBV)	none
						pSauR222-2	2,422	JAIUEB010000049.1	2267.4	rep10 (RepL)	none	ermC
SauR223	2019/ pus	NA	JAIWLW000000000	387.6	FOX,P,E, DA,TE; MDR (4 classes); iMLSB	pSauR223-1	21,497	JAIWLW010000017.1	1614.4	rep20 (RepA_N) + rep5a (Rep_3) +Δrep21(ΔRep_1)	Mob/pre (MOBV)	bla operon
						pSauR223-2	4,534	JAIWLW010000020.1	1627.5	rep7a (Rep_trans)	Mob/pre (MOBV)	tetK
						pSauR223-3	2,473	JAIWLW010000022.1	1345.7	rep10 (RepL)	none	ermC
SauR224	2019/ Nasopharyngeal	CC22	JAIXHF000000000	410.4	FOX, OX, P, CFP, CIP, E, DA; MDR (4 classes); iMLSB	pSauR224-1	3011	JAIXHF010000034.1	2089.6	rep21 (Rep_1)	pWBG749 oriT-OT49 mimic + rep-rlx	none
						pSauR224-2	2,473	JAIXHF010000036.1	1568.0	rep10 (RepL)	none	ermC
SauR225	2019/ Respiratory	CC22	JAIUDX010000000	389.6	FOX, OX, P, CIP, MXF, E, DA; MDR (4 classes); iMLSB	pSauR225-1	35,336	JAIUDX010000030.1	1619.0	rep20 (RepA_N) + rep5a (Rep_3)	Mob/pre (MOBV)	none
						pSauR225-2	3,050	JAIUDX010000044.1	1410.3	rep21 (Rep_1)	pWBG749 oriT-OT49 mimic + rep-rlx	none
						pSauR225-3	2,528	JAIUDX010000045.1	999.4	rep10 (RepL)	none	ermC
SauR235	2019/ pus	CC22	JAIUDY010000000	439.2	FOX, OX, P, CIP, E, DA; MDR (4 classes); iMLSB	pSauR235-1	35,501	JAIUDY010000032.1	1793.1	rep20 (RepA_N) + rep5a (Rep_3)	Mob/pre (MOBV)	none
						pSauR235-2	2,528	JAIUDY010000050.1	1497.3	rep10 (RepL)	none	ermC
SauR248	2019/ pus	NA	JAIXHG000000000	381.1	FOX, P, CIP, MXF, CN; MDR (3 classes)	no plasmid detected	-	-	NA	-	-	-
SauR253	2020/ pus	NA	JAIWLX000000000	374.5	FOX, OX, P, CIP, MXF, CN, TGC; MDR (4 classes)	pSauR253	20,727	JAIWLX010000018.1	1251.9	rep5a (Rep_3)	Mob/pre (MOBV)	bla operon
SauR255	2020/ urine	NA	JAKRCN000000000	349.3	FOX, OX, P, CIP, MXF, E, DA, CN, TGC. MDR (5 classes); iMLSB	pSauR255	20,727	JAKRCN010000013	1589.5	rep5a (Rep_3)	Mob/pre (MOBV)	bla operon

*Abbreviations used: **β-lactams:** penicillin, P; oxacillin, OX; cefoxitin, FOX; cefoperazone, CFP; **fluoroquinolones**: ciprofloxacin, CIP; moxifloxacin, MXF; **macrolides:** erythromycin, E; **lincosamides:** clindamycin, DA; **aminoglycosides:** gentamicin, CN; amikacin, AK; **folate inhibitors:** co-trimoxazole, SXT; **fucidanes,** fusidic acid, FD; **tetracyclines:** tetracycline, TE; doxycycline, DO; minocycline, MH; **glycylcyclines:** tigecycline, TGC; **phenicols:** chloramphenicol, C; **monocarboxylic acid:** mupirocin, MUP; **ansamycins:** rifampin, RD; **aminocoumarin:** novobiocin, NV; **glycopeptides:** vancomycin, VA; teicoplanin, TEC; **oxazolidinones:** linezolid, LZD; **phosphonic acids**: fosfomycin, FOT; **stretogramins:** quinupristin-dalfopristin, QD; **anti-MRSA cephalosporins:** ceftaroline, CPT. **MLS_B phenotypes**: inducible (I), iMLSB; constitutive (C), cMLSB; macrolide-streptogramin, MS. MDR, multidrug resistance (resistance ≥ 3 classes of antimicrobials).

Strain code	Year of isolation / Source of strain	Clonal Complex (CC)	Strain accession no.	Chromosome coverage (average)	Resistance phenotype*	Plasmid name	Size of plasmid in bp	Plasmid accession no.	Plasmid coverage (average)	Replicon type (Conserved domain)	Mobility mechanism(s)	Antimicrobial resistance determinant(s)
SauR257	2020/ blood	NA	JAIXAI000000000	431.2	FOX, OX, P, CIP, CN, MXF; MDR (3 classes)	pSauR257	20,727	JAIXAI010000017.1	1984.4	rep5a (Rep_3)	Mob/pre (MOBV)	bla operon
SauR258	2020/ blood	NA	JAIXAJ000000000	457.7	FOX, OX, P, CIP, MXF, CN, TGC. MDR (4 classes)	pSauR258	20,728	JAIXAJ010000017.1	1525.5	rep5a (Rep_3)	Mob/pre (MOBV)	bla operon
SauR261	2020/ blood	NA	JAIXAK000000000	445.5	FOX, OX, P, CIP, FD; MDR (3 classes)	pSauR261	20,728	JAIXAK010000013.1	1880.8	rep5a (Rep_3)	Mob/pre (MOBV)	bla operon
SauR262	2020/ pus	CC5	JAKRCO000000000	419.6	FOX, P, E, TGC; MDR (3 classes); MS	pSauR262	14,935	JAKRCO010000017	1236.5	repUS23 (RepA_N)+ repUS46 (rep_2)	none	none
SauR263	2020/ blood	CC22	JAKRCP000000000	381.5	FOX, OX, P, CIP, CFP, MXF, E, DA; MDR (4 classes); iMLSB	pSauR263-1	35,334	JAKRCP010000082	1770.5	rep20 (RepA_N) + rep5a (Rep_3)	Mob/pre (MOBV)	none
						pSauR263-2	3,050	JAKRCP010000049	1342.5	rep21 (Rep_1)	pWBG749 oriT-OT49 mimic + rep-rlx	none
						pSauR263-3	2,528	JAKRCP010000051	1178.9	rep10 (RepL)	none	ermC
SauR265	2020/ blood	CC22	JAKRCQ000000000	395.0	FOX, OX, P, CIP, MXF, E, DA; MDR (4 classes); iMLSB	pSauR265-1	3,011	JAKRCQ010000032	1380.3	rep21 (Rep_1)	pWBG749 oriT-OT49 mimic + rep-rlx	none
						pSauR265-2	2,418	JAKRCQ010000034	1180.2	rep10 (RepL)	none	ermC
SauR266	2020/ blood	CC22	JAIXHH000000000	404.5	FOX, OX, P, CIP, E, DA; MDR (4 classes); iMLSB	pSauR266	2,462	JAIXHH010000029.1	1887.5	rep10 (RepL)	none	ermC
SauR268	2020/ blood	CC22	JAIUDZ010000000	343.8	FOX, OX, P, CIP, E, DA; MDR (4 classes); iMLSB	pSauR268-1	35,336	JAIUDZ010000029.1	1560.3	rep20 (RepA_N) + rep5a (Rep_3)	Mob/pre (MOBV)	none
						pSauR268-2	3,050	JAIUDZ010000042.1	5045.9	rep21 (Rep_1)	pWBG749 oriT-OT49 mimic + rep-rlx	none
						pSauR268-3	2,528	JAIUDZ010000043.1	4007.9	rep10 (RepL)	none	ermC
SauR269	2020/ pus	CC22	JAKRCR000000000	251.6	FOX, OX, P, CIP, MXF, E, DA; MDR (4 classes); iMLSB	pSauR269-1	35,332	JAKRCR010000170	1025.8	rep20 (RepA_N) + rep5a (Rep_3)	Mob/pre (MOBV)	none
						pSauR269-2	14,935	JAKRCR010000051	144.9	repUS23 (RepA_N)+ repUS46 (Rep_2)	none	none
						pSauR269-3	2,473	JAKRCR010000081	1107.4	rep10 (RepL)	none	ermC

*Abbreviations used: **β-lactams:** penicillin, P; oxacillin, OX; cefoxitin, FOX; cefoperazone, CFP; **fluoroquinolones**: ciprofloxacin, CIP; moxifloxacin, MXF; **macrolides:** erythromycin, E; **lincosamides:** clindamycin, DA; **aminoglycosides:** gentamicin, CN; amikacin, AK; **folate inhibitors:** co-trimoxazole, SXT; **fucidanes,** fusidic acid, FD; **tetracyclines:** tetracycline, TE; doxycycline, DO; minocycline, MH; **glycylcylines:** tigecycline, TGC; **phenicols:** chloramphenicol, C; **monocarboxylic acid:** mupirocin, MUP; **ansamycins:** rifampin, RD; **aminocoumarin:** novobiocin, NV; **glycopeptides:** vancomycin, VA; teicoplanin, TEC; **oxazolidinones:** linezolid, LZD; **phosphonic acids**: fosfomycin, FOT; **stretogramins:** quinupristin-dalfopristin, QD; **anti-MRSA cephalosporins:** ceftaroline, CPT. **MLS_B phenotypes**: inducible (I), iMLSB; constitutive (C), cMLSB; macrolide-streptogramin, MS. MDR, multidrug resistance (resistance ≥ 3 classes of antimicrobials).

Genome data of other Malaysian MRSA strains retrieved from GenBank:

Strain code	Year of isolation/ Source of strain	Clonal Complex (CC)	Strain accession no.	Chromosome coverage (average)	Resistance phenotype*	Plasmid name	Size of plasmid in bp	Plasmid accession no.	Plasmid coverage (average)	Replicon type (Conserved domain)
SAZ_1	2012 / body fluid from infection	CC8	BioProject : PRJNA503680	PEN, FOX, OX	pSAZ_1-1	29,265	multi-contigs: SDOB01000025.1 SDOB01000029.1 SDOB01000032.1 SDOB01000039.1 SDOB01000050.1	rep20 (RepA_N) + rep21 (Rep_1)	pWBG749 oriT-OTUNa+ pWBG749 oriT-OT49 mimic + rep-rlx	none
					pSAZ_1-2	4,442	SDOB01000031.1	rep7a (Rep_trans)	Mob/pre (MOBV)	cat
SAZ_31	2013 / pus	CC30	BioProject : PRJNA503680	PEN, FOX, OX, NOR, CN, CIP, FD	pSAZ_31-1	24,749	multi-contigs: SDOE01000022.1 SDOE01000026.1	rep19 (RepA_N) + rep21 (Rep_1)	pWBG749 oriT-OT49 mimic + rep-rlx	bla operon
MRSA SAZ_10	2012 / pus	CC8	BioProject : PRJNA503680	PEN, FOX, OX, NOR, DA, E; iMLSB	pSAZ10A	35,123	SWED01000025.1	rep15 (RepA_N)	conjugative	ant(4')-Ib, mupA
					pSAZ10B	29,214	multi-contigs: SWED01000032.1 SWED01000035.1 SWED01000038.1 SWED01000045.1 SWED01000046.1 SWED01000055.1	rep20 (RepA_N) + rep21 (Rep_1)	pWBG749 oriT-OTUNa+ pWBG749 oriT-OT49 mimic + rep-rlx	none
					pSAZ1C	2,736	SWED01000040.1	rep10 (RepL)	none	ermC
ZS_Z30	2016 / nasal swab	CC8	BioProject : PRJNA503680	PEN, FOX, OX, TE, CN, DA, SXT, E, CIP; cMLSB	pZS_Z30-1	21,173	multi-contigs: RZHI01000026.1 RZHI01000033.1 RZHI01000042.1	rep20 (RepA_N) + rep21 (Rep_1)	pWBG749 oriT-OTUNa+ pWBG749 oriT-OT49 mimic + rep-rlx	none
					pZS_Z30-2	2,723	RZHI01000036.1	rep21 (Rep_1)	rep-rlx	none
ZS-Z37	2016 / nasal swab	CC8	BioProject : PRJNA503680	PEN, FOX, OX, TE, CN, SXT, E, CIP; iMLSB	pZS-Z37-1	26,250	multi-contigs: RZHJ01000023.1 RZHJ01000036.1 RZHJ01000060.1	rep20 (RepA_N) + rep21 (Rep_1)	pWBG749 oriT-OTUNa+ pWBG749 oriT-OT49 mimic + rep-rlx	none
					pZS-Z37-2	4,714	RZHJ01000039.1	rep7a (Rep_trans)	MOBV/Mob_Pre	tetK

*Abbreviations used: **β-lactams:** penicillin, P; oxacillin, OX; cefoxitin, FOX; cefoperazone, CFP; **fluoroquinolones**: ciprofloxacin, CIP; moxifloxacin, MXF; **macrolides:** erythromycin, E; **lincosamides:** clindamycin, DA; **aminoglycosides:** gentamicin, CN; amikacin, AK; **folate inhibitors:** co-trimoxazole, SXT; **fucidanes,** fusidic acid, FD; **tetracyclines:** tetracycline, TE; doxycycline, DO; minocycline, MH; **glycylcylines:** tigecycline, TGC; **phenicols:** chloramphenicol, C; **monocarboxylic acid:** mupirocin, MUP; **ansamycins:** rifampin, RD; **aminocoumarin:** novobiocin, NV; **glycopeptides:** vancomycin, VA; teicoplanin, TEC; **oxazolidinones:** linezolid, LZD; **phosphonic acids**: fosfomycin, FOT; **stretogramins:** quinupristin-dalfopristin, QD; **anti-MRSA cephalosporins:** ceftaroline, CPT. **MLS_B phenotypes**: inducible (I), iMLSB; constitutive (C), cMLSB; macrolide-streptogramin, MS. MDR, multidrug resistance (resistance ≥ 3 classes of antimicrobials).

Supplementary Table S1

Strain code	Year of isolation/ Source of strain	Clonal Complex (CC)	Strain accession no.	Chromosome coverage (average)	Resistance phenotype*	Plasmid name	Size of plasmid in bp	Plasmid accession no.	Plasmid coverage (average)	Replicon type (Conserved domain)
A54_B	2016 / nasal swab	NA	BioProject : PRJNA503680	PEN, TE	no plasmid detected	-	-	-	-	-
SAZ_16	2013 / blood	CC1		PEN, FOX, OX, NOR, CN, CIP, FD	no plasmid detected	-	-	-	-	-
SAZ_10	2012/ pus	CC8		PEN, FOX, OX, NOR, DA, E (iMLSb)	no plasmid detected	-	-	-	-	-
ZS_Z46	2016 / nasal swab	CC8		PEN, FOX, OX, TE, CN, SXT, E, CIP; iMLSB	no plasmid detected	-	-	-	-	-
KT/Y21	2009 / blood	CC1	AOCQ00000000.1	PEN, FOX, OX, FD, MUP, N, KAN, CN	no plasmid detected	-	-	-	-	-
MRSA_PR 1	2009 / blood	CC8	ANPO00000000.1	unavailable	no plasmid detected	-	-	-	-	-
PPUKM-261-2009	2009 / tracheal aspirate	NA	AMRB00000000.1	unavailable	pPPUKM-261-2009-1	25,327	multi-contigs: AMRB01000083.1 AMRB01000003.1 AMRB01000078.1 AMRB01000086.1 AMRB01000082.1 AMRB01000091.1 AMRB01000092.1 AMRB01000093.1 AMRB01000094.1	rep20 (RepA_N)	pWBG749 oriT-OUNa+ pWBG749 oriT-OT49 mimics	none
PPUKM-332-2009	2009 / tracheal aspirate	CC8	AMRC00000000.1	unavailable	pPPUKM-332-2009-1	24,010	multi-contigs: AMRC01000117.1 AMRC01000113.1 AMRC01000114.1 AMRC01000115.1 AMRC01000118.1 AMRC01000124.1	rep20 (RepA_N)	pWBG749 oriT-OTUNa+ pWBG749 oriT-OT49 mimics	none
					pPPUKM-332-2009-2	4,260	AMRC01000004.1	rep7a (Rep_trans)	MOBV/Mob_pre	cat
PPUKM-377-2009	2009 / tracheal aspirate	CC5	AMRD00000000.1	unavailable	pPPUKM-377-2009-1	28,544	multi-contigs: AMRD01000082.1 AMRD01000084.1 AMRD01000086.1 AMRD01000091.1 AMRD01000092.1 AMRD01000094.1 AMRD01000079.1	rep19 (RepA_N) + rep20 (RepA_N)	pWBG749 oriT-OTUNa+ pWBG749 oriT-OT49 mimics	none
					pPPUKM-377-2009-2	2,478	AMRD01000090.1	rep10 (repL)	none	ermC

*Abbreviations used: **β-lactams:** penicillin, P; oxacillin, OX; cefoxitin, FOX; cefoperazone, CFP; **fluoroquinolones**: ciprofloxacin, CIP; moxifloxacin, MXF; **macrolides:** erythromycin, E; **lincosamides:** clindamycin, DA; **aminoglycosides:** gentamicin, CN; amikacin, AK; **folate inhibitors:** co-trimoxazole, SXT; **fucidanes,** fusidic acid, FD; **tetracyclines:** tetracycline, TE; doxycycline, DO; minocycline, MH; **glycylcylines:** tigecycline, TGC; **phenicols:** chloramphenicol, C; **monocarboxylic acid:** mupirocin, MUP; **ansamycins:** rifampin, RD; **aminocoumarin:** novobiocin, NV; **glycopeptides:** vancomycin, VA; teicoplanin, TEC; **oxazolidinones:** linezolid, LZD; **phosphonic acids**: fosfomycin, FOT; **stretogramins:** quinupristin-dalfopristin, QD; **anti-MRSA cephalosporins:** ceftaroline, CPT. **MLS_B phenotypes**: inducible (I), iMLSB; constitutive (C), cMLSB; macrolide-streptogramin, MS. MDR, multidrug resistance (resistance ≥ 3 classes of antimicrobials).

Strain code	Year of isolation/ Source of strain	Clonal Complex (CC)	Strain accession no.	Chromosome coverage (average)	Resistance phenotype*	Plasmid name	Size of plasmid in bp	Plasmid accession no.	Plasmid coverage (average)	Replicon type (Conserved domain)
PPUKM-775-2009	2009 / tracheal aspirate	CC8	AMRE00000000.1	unavailable	pPPUKM-775-2009-1	20,294	multi-contigs: AMRE01000085.1 AMRE01000089.1 AMRE01000080.1 AMRE01000096.1 AMRE01000097.1	rep20 (RepA_N)	pWBG749 oriT-OTUNa mimic	none

*Abbreviations used: **β-lactams:** penicillin, P; oxacillin, OX; cefoxitin, FOX; cefoperazone, CFP; **fluoroquinolones**: ciprofloxacin, CIP; moxifloxacin, MXF; **macrolides:** erythromycin, E; **lincosamides:** clindamycin, DA; **aminoglycosides:** gentamicin, CN; amikacin, AK; **folate inhibitors:** co-trimoxazole, SXT; **fucidanes,** fusidic acid, FD; **tetracyclines:** tetracycline, TE; doxycycline, DO; minocycline, MH; **glycylcylines:** tigecycline, TGC; **phenicols:** chloramphenicol, C; **monocarboxylic acid:** mupirocin, MUP; **ansamycins:** rifampin, RD; **aminocoumarin:** novobiocin, NV; **glycopeptides:** vancomycin, VA; teicoplanin, TEC; **oxazolidinones:** linezolid, LZD; **phosphonic acids**: fosfomycin, FOT; **stretogramins:** quinupristin-dalfopristin, QD; **anti-MRSA cephalosporins:** ceftaroline, CPT. **MLS_B phenotypes**: inducible (I), iMLSB; constitutive (C), cMLSB; macrolide-streptogramin, MS. MDR, multidrug resistance (resistance ≥ 3 classes of antimicrobials).

Supplementary Table S2: List of primers used for gap closures and sequence validation of identified plasmids.

**Primer sequences (5′→3′)**	**Description**
F: ACGCTTTATAAAATGCATCGAACAC<br>R: TGTCGTACTCGTCATTTGACC	Used to validate pSauR127 and pSauR209 sequences
F: CCAAGACAGCTCAAACTAATACCAAT<br>R: CATTTATGGCGTGTAGAAATCGAAC	Used to validate pSauR7 and pSauR76-2 sequences
F1: GTCCAATTTGACATAGAGCCTTGATC<br>R1: CTGTTAATGGTTCTGATGGGCAG	Used to close and validate gaps in the 35 kb heavy metal plasmids.
F2: CCTGCCCATCAGAACCATTAACAG<br>R2: CAGTTGATCGTGCGTTCACCT	
F3: GCGAACATCACGTTTGCCATC<br>R3: GACGTTATATCTCTGGAACCTGGAC<br>Internal-F3: AGAGTAAATCCTAATACTG	Used to close and validate gaps in pSauR47-1, pSauR77-1, pSauR81-1, pSauR82-1, pSauR105-1, pSauR106-1, pSauR143-1, and pSauR142-1.
F4: GCAGCTTTAGCTTTACATACTCTCG<br>R4: GATCAAGGCTCTATGTCAAATTGGAC	Used to close gaps in pSauR47-1.
F1: GCAATCGCAACGATAAATAG<br>R1: GGACAAAATGTGATTTAAATGAC	Used to close gaps in pSauR76-1
F2: GAGCGTACACATGAAACTTTC<br>R2: TACGACTGATGAGAGTGAAG	
F3: GCTTCCCAATCCTCCTATCA<br>R3: GGTTCAAGACATTCAAGGAAATC	.
F: AGTCGTTAGAAGCTCATATTTGCG<br>R: AGAGAGATGTCACCGTCAAG	Used to validate pSauR156-1 sequence.
F: AGATGGGTGTTTGGAGTTATCG<br>R: AGCTACTGCAATTAACGCAATC	Used to validate pSauR23-1 sequence.
F1: ACTTGAAGATAGTGAAGAAGAAATAGG<br>R1: AGGGTAATTTAAATGGCGAATGG	Used to close and validate gaps in pSauR165-1.
F2: AAGGGTGTGCTTAAATCGGG<br>R2: ACCGTCATCTTCCATGTAGC	
F3: TACGAAAATGCAAAGCCACT<br>R3: AGCAGCAGTTACAATAGTCTGAATC	
F4: ATGCAAATGAGATCTTCTCACAGC<br>R4: ACTTCTTCCATAAACTCAGTCAAGTCC	
F5: GTTATTGGTTTGTTATCAATTCC<br>R5: GTCATAGTTTGTGAAAACTAAGTT	
F1: AGTGAGAACTTCTGGTAATCATC<br>R1: AGCCAACTATACTTTTATTCCCG	Used to close and validate gaps in pSauR3-1, pSauR90, and pSauR169
F2: AGCCTGTACACGACTGATAG<br>R2: ACTCCAGTAATATGATTAAGGTGTC	
F3: TAGACTTGAAACCGTTGATATCACAG<br>R3: TCACTGAAAGATAAAATTCCTAAGC	
F: TGAATCGTTGATATGACGGCG<br>R: ATACACCTCCTCTCTGCGTC	Used to validate pSauR223-1 sequences
F: AGAGTGTGTCATTACTTTAGACTG<br>R: CCATGTGTATGATTATGATTGTGATCGT	Used to validate pSauR262 and pSauR269-2 sequences
